# Spirit of the Foreign Periodicals, &c.

**Published:** 1840

**Authors:** 


### Spirit of the Foreign Periodicals.

### M. Lisfranc on the Necessary Alliance between Medicine and Surgery.

"Of late years these two divisions of the healing art have contracted an indissoluble alliance. It is impossible to be a truly good surgeon without being a good physician also; or to be a good physician without having practical knowledge of surgical maladies. Who would think in the present day of amputating a limb, without having previously examined, with most minute care, the state of all the visceral organs? There may be a tubercular state of the lungs, or a latent inflammation of the intestinal tube, which needs only the general reaction, which necessarily follows every great operation, to explode with violence. How then can you guard against such mischief, unless you possess the knowledge requisite to detect the existence of these morbid states? The neglect of ascertaining the state of the inward organs, before having recourse to the use of the knife, has been the main cause of operative surgery having so often fallen into bad repute. Again, in severe injuries of the head the chief danger, it is well known, arises from sympathetic disease induced in the brain or its membranes."

The following censure is liberal on the part of a Frenchman, as it exposes—we need not say, very deservedly and truly—the prevailing errors of his fellow-countrymen.

. . . . "A most pernicious heresy has sprung up among medical men of late years;—to wit, that 'the nature of a disease being known, its treatment is so likewise.' As if it was sufficient to know the mere name of any weapon to be able to use it aright. This error has arisen chiefly from the unwise attempt to reduce under general rules numerous morbid individualities, each of which constitutes, so to speak, a separate exception. Thus, it has no doubt been the cause of those absurd systems of *exclusivism* in medical practice which have been so frequently brought forward—one set of men lauding on all occasions the use of the lancet, another the use of purgatives, a third of the chlorurets, and a fourth of tonics, in the treatment of one and the same disease. The history of medicine exhibits, it must be confessed, anything but a creditable picture of the professional sagacity, or even of the common sense, of its professors. One quarter of a century has very often been almost solely occupied in overthrowing what its predecessor had been engaged in building up; and such will ever continue to be the fate of physic, until medical men are properly impressed with the truth, that every case of a disease presents some individual difference or peculiarity—arising from a multitude of causes, such as constitution, the state of the weather, the condition of the mind and feelings, &c. &c.

"I conjure you," adds M. *Lisfranc*, "to avoid being misled by opposite doctrines; such as you will hear professed elsewhere."

(This is a hit, we suppose, at M. *Bouillaud* and other followers of the *Broussais* regime, as well as at several other of the academicians who have been of late discussing the utility of purgatives in typhus.)

*Absurd Practice in some of the Paris Hospitals.*

M. *Lisfranc*, still commenting on the dangerous errors of *exclusivism* in medicine and on the extravagancies of certain modern reformers, continues.

"Just look at the practice of some of these gentlemen in their hospitals! See them ordering the same dose of a medicine to twenty different patients, affected with the same malady; and the administration is entrusted entirely to some young inexperienced pupil, who cannot be expected to know how to accommodate a remedy to different cases. Then, according to the report made to him, the physician takes upon himself to commend or to condemn the treatment that has been pursued."

Such a *tableau,* drawn too by one of the leading hospital surgeons in Paris, strongly confirms the truth of the opinions which the *Medico-Chirurgical Review* has uniformly professed in reference to French medical practice. Every one has heard of the satire in one of *Moliere's* comedies, where the Doctor is represented as ordering all the patients on one side of a hospital ward to be bled, and all those on the other side to be purged, without reference to their maladies; and here we have the assurance of such a man as M. *Lisfranc* that the picture is not very inapplicable to some of his confreres at the present moment in the French metropolis.

"Truth is strange, stranger than fiction."

—*La Lançette Française.*

---

M. LISFRANC ON SOME OF THE DISEASES OF THE MAMMA.

. . . . . . . "Women will come to you complaining of pains in their breasts; you examine them, but finding no mark of swelling, heat, or redness in the parts, you may be tempted to tell them that there is very little the matter with them. Be on your guard; the mischief may exist elsewhere, and the mammæ may be only sympathetically suffering. Direct your attention to the state of the uterus; and always bear in mind that each menstrual effort determines a consentaneous increase of vital energy in the mammæ: in some cases, this increased sensibility exists from one monthly period to another, and will give rise to constant sharp pains in the glands.

By relieving the existing uterine irritation, the distress in the mammæ will speedily subside.

In another set of cases, however, these pains in the mammæ may arise from a neuralgic affection of their own nerves, quite unconnected with any uterine ailment.

Now, although there may be no signs of inflammatory action present, the surgeon should never neglect the relief of these neuralgic pains; more especially as the very existence of severe pain in any part, and especially if that part be of a glandular texture, is apt to induce an insidious chronic excitement of the capillary vessels, which may ultimately terminate in morbid structure.

Let us now consider another set of cases, in which the symptoms are again different.

"A woman, without having received any blow or injury on her breasts, may experience in one or in both of them a dull heavy pain, which is more or less severe at times, but which seldom ceases entirely: its chief seat exists usually at one spot, which, however, exhibits no visible signs of engorgement or inflammation. What are we to think of such a case, which is of very frequent occurrence? We should suspect that there exists, either in the granulations of the gland or in its cellular tissue, a chronic inflammatory action, which is, as it were, still latent, but which if neglected will almost inevitably lay the foundation of some serious degeneration of tissue.

We have said that such a state of things may come on without having been preceded by any external violence to the mamma. In some cases however, as might be imagined, it follows upon a blow or other injury—the immediate effects of which, having been removed at the time by leeches, &c. may have been entirely forgotten. A fixed pain continues, nevertheless, in one point, felt perhaps only on pressure; it is usually more troublesome at each catamenial period; and yet there may be no visible engorgement or hardness even then. In such a case as this, be assured that there exists a latent chronic inflammation. I have repeatedly seen engorgements in the first place, and subsequently degenerations of structure, supervene, after the lapse of three, six, or twelve months, in women who had neglected to use the means which I had recommended at the time. The use of over-tightly laced stays, which keep up an unnatural compression of the mammæ, is a not unfrequent cause of permanent mischief."

"In the treatment of chronic tumours of the mamma, the first thing to do is to ascertain whether there be any latent and disguised inflammatory action in or around the swelling. This may generally be easily determined by observing the effects of pressure with the fingers. Whenever there is decided pain, even although this be only of a dull character, we may apprehend the existence of vascular excitement in the part. Under such circumstances the application, to be repeated in 8 or 10 days if necessary, of leeches and then of laudanised poultices, is the most important remedy. The arm of the affected side should be constantly suspended in a sling; the diet should consist of light food; and tepid baths should be used twice or thrice a week.

The practice of smearing the mamma with a thick layer of strong mercurial ointment, to be renewed four or five times in the twenty-four hours, and to be continued steadily for a few days, as recommended by M. *Serres*, is approved of by M. *Lisfranc*. He also recommends the internal use of certain depurative decoctions, made with saponaria, scabiosa, and some bitter roots, and also of small doses of hemlock—the powder of which, in the dose of a grain at first to be rapidly increased, he greatly prefers to the extract.

When all traces of inflammatory action have quite ceased, we should then commence the employment of discutient means. *Compression* by means of strapping and of bandages, &c. is one of the best of these. It is a remedy, however, that should be used with great caution at first; as it is very apt to re-excite inflammatory action.

*Douches*, of simple and afterwards of aromatised vapour, have sometimes the most pleasing effects on indolent tumors of the mammæ.

*Friction* with various ointments and embrocations—containing the ioduret of lead, the hydriodate of ammonia and of potash, various preparations of mercury, &c.—may often be used at the same time with advantage. Indeed, in the management of most chronic complaints, it is a most useful practice to combine the use of various means at one and the same time. Thus in the present set of cases, the vapour-douches, compression, and friction may be all employed together, not omitting the administration of appropriate remedies internally, of which the hydriodate of potash in conjunction with bitter infusions is perhaps one of the best."

The following remarks of M. *Lisfranc* on the advantages of local bleedings in cases of genuine scirrhous and cancerous disease are of practical importance.

"Twenty years ago, it was pretended that cancer might be cured by antiphlogistic remedies. M. *Lallemand*, of Montpellier, published several cases of cancer of the lip as cured by these means; *Broussais* also narrated some similar cases, and I myself added a few.

But cancer of the skin is not cancer of the cellular, and far less that of the glandular, tissue. Nevertheless I attempted to cure ulcerated cancer of other parts without having recourse to extirpation of the diseased parts; and in these attempts I often observed that the ulceration in the centre of the diseased swelling became for a time arrested, and as it were withered, but speedily began to spread as before, while the amelioration of the circumference was progressive and permanent. At that time I was content with merely observing and recording the fact, without endeavouring to explain it; but gradually opportunities of more attentively examining the pathological phenomena in scirrhous and cancerous tumours were afforded me. The following was the result of these investigations. In the circumferential part of the tumour I found all the usual phenomena of pure inflammation ; nearer the centre there was simple white induration ; still nearer, this white induration was observed to have passed into the state of scirrhus ; and in the centre itself was the genuine cancerous degeneration. Now then, the judicious employment of leeches and other antiphlogistic remedies may succeed in removing all the circumferential morbid changes, although we cannot hope that they will dissipate, or even arrest, the central degeneration. This, however, is an important end gained; for in this manner we may reduce the size of the swelling to one half of its former size, and thus render the operation, which is necessary, less formidable and severe independently of the surrounding parts being at the same time brought into a state which is more healthy and more disposed to speedy union and cicatrisation. I again repeat, however, that we must not expect, in any case, to get rid of the central or genuine cancerous tumor by the use of antiphlogistic or of any other local means ; and I have seen much mischief follow from an unwisely prolonged attempt to effect this."

In a patient, whom M. L. recently saw, the swelling of the mamma had been reduced by at least two thirds, and then it became and remained stationary; nevertheless, she was recommended by her medical attendants to persevere with the use of leeches, &c. For a month no additional progress had been made; and therefore M. *Lisfranc*, who was now called in, advised the immediate extirpation of the tumor. Unfortunately the lady was induced, by the suggestion of some friends, to defer submitting to it for several weeks; a *cancerous explosion* took place, several new ulcerated points formed, and the patient died.

As a general rule, M. *Lisfranc* recommends that the operation should not be delayed, whenever there has been no visible amendment of the swelling for three or four weeks past. If such be the case, it is not only a fruitless annoyance of the patient to continue the use of leeches and other antiphlogistic remedies, but the cancerous disease may thereby be permitted to make serious progress, and thus render the removal of the tumor more difficult and severe.—*La Lançette Française.*

---

### ON THE INFLUENCE OF SPLENIC ENGORGEMENTS ON AGUES.

It is quite unnecessary to do more than simply allude to the very great frequency of congestion and enlargement of the spleen after attacks of intermittent fever. The important question is, what is the effect of such a state on the health of the patient? and how is it best remedied?

M. *Bally* was the first to shew distinctly that the marked tendency to the relapse of intermittent fevers, after they have been seemingly got rid of, is mainly attributable to the engorgement of the spleen which is permitted to remain. The fever may have quite ceased to return for one or even several months, but the patient, notwithstanding, does not recover his former health and strength ; his complexion remains pale, his appetite is variable, his digestion is not good, and his muscular energy remains feeble ; and at length the periodical feverish symptoms begin again to make their appearance.

In a recent lecture at the Hôpital de la Charité, M. *Nonat* has inculcated the opinions of his predecessor with much ability.

"We will not affirm," says he, "that hypertrophy of the spleen produces intermittent fever ; this has its *point de depart* in some unknown cause, whether of the nature of miasm or otherwise, and inappreciable in its nature ; but certainly it cannot be denied that such hypertrophe is an occasional and predisposing cause, which keeps the system under the influence of this primitive agent."

By far the most potent agent in subduing this enlarged state of the spleen is the Peruvian bark. But this must be given in larger doses than is usually done. It is more than probable that the frequency of the relapse of agues is mainly attributable to the insufficient administration of this most valuable specific. Such was the opinion of *Morton, Sydenham* and *Torti;* and the experience of the best practitioners in the present day confirms its truth.

M. *Nonat* remarks: "M. *Bally* was the first in France who distinctly pointed out the efficacy of quinine in the treatment of intermittent fevers, and more especially in those cases which are accompanied with considerable engorgement of the spleen. According to this experienced practitioner, we should commence with eight or ten grains of the salt every eight hours, and raise the dose quickly, if the case be obstinate, to 40 or even 60 grains in the course of the day. I have seen M. *Bally* obtain, by this method, a success which it is vain to expect from the same remedy when administered in the usual manner."

M. *Piorry* assures us that under the use of large doses of quinine—from 40 to 80 grains in the course of twenty-four hours—he has seen considerable engorgements of the spleen subside in a few days.*

M. *Nonat* has investigated this subject of practical medicine with much zeal: and the conclusion at which he has arrived is, that the quantity of quinine required to effect a permanent cure of an ague, and of the induced enlargement of the spleen, is almost always in a direct ratio with the amount or extent of this enlargement. "After a great many experiments," he says, "I have satisfied myself that the dose of quinine should vary from 12 to 40 or 50 grains in the course of twenty-four hours, and that it must be proportioned to the increase in the size of the spleen; such are the doses which we have been in the habit, for the last three years, of using with such marked success. Hitherto we have met with very few cases indeed of enlargement of the spleen, which have resisted this method of employing the quinine."

The length of time, during which it may be necessary to continue the use of the remedy, varies much according to several circumstances, such as the extent of the enlargement, the duration of the complaint, the constitution of the patient, and so forth. But, whatever be the case, its employment should not be discontinued until the viscus has resumed its normal dimensions. In some cases, the stomach will not bear the large doses that are required; we must then administer the quinine in enemata, and in still greater quantities. To derive success from this mode of treatment, it is quite necessary that the enemata be retained; else all the febrifuge effects of the quinine will not be obtained. "When this condition is fulfilled," says M. Nonat, "I have in every case observed that the fever ceased, and the engorgement of the spleen subsided, as quickly as when the quinine had been administered by the mouth; and this too in patients affected with most obstinate agues which had resisted the ordinary plan of treatment in various hospitals."

M. *Nonat* does not expect much from the endermic use of quinine, as recommended by some physicians: the fever may indeed be cured, but not the splenic disease, in this way. He has tried in a few instances the *iatroleptic* method, or that of rubbing the quinine in, in the form of an ointment. Forty or fifty grains of the salt were mixed with two ounces of lard, and the patient was directed to rub in some of this ointment on the groins and armpits three times a day. Under this mode of treatment, the fever has been known to cease, and

* Dr. *Elliotson,* in some of his clinical lectures reported in the Lancet five or six years ago, very forcibly inculcated the same views as to the treatment of the enlargements of the spleen, which so frequently follow intermittent fevers." —*Rev.*

the splenic enlargement to subside a little; but never to any considerable extent, or permanently.

Before closing these remarks, we should mention that, in most cases of ague attended with enlargement of the spleen, the local abstraction of blood, by cupping or leeches, from the left hypochondrium will greatly promote the efficacy of the quinine.—*La Lançette Française.*

---

### M. CAZENAVE ON SCABIES.

The following extracts are from a lecture which M. *Cazenave* recently delivered at the hospital St. Louis, to which, it is well known, by far the greater number of patients affected with cutaneous diseases in Paris are sent.

"With respect to the seat of, or the parts of the body most frequently affected with, the itch, it is right to observe that certain conditions and occupations of life, under the influence of which the disease is apt to be developed, induce varieties which deserve to be noticed. Thus in smiths and dyers we rarely observe the scabious eruption on the wrists or between the fingers; whereas these are just the parts which are almost always affected in tailors and semstresses—who constitute a large proportion of itch-patients admitted into the Hospital St. Louis. M. *Cazenave* has never seen the face affected with the disease."

. . . . . "When the disease is left to itself, more especially in young plethoric persons, it is apt to become complicated with other forms of cutaneous eruption—most frequently of the impetiginous or ecthymatous character. It is from not being aware of this complication, that some writers have described scabies as a pustular disease."

. . . . . "It is now universally admitted that the proximate cause of the itch is the presence of an animalcule—the *acarus scabiei*. It is well known how frequently the truth of this idea has been questioned. The cause of the difficulty of detecting the insect was first explained by M. *Renucci* in 1834, who shewed that it is seldom found in the vesicle itself, but generally in the groove of the skin leading from it, and which the insect itself makes under the epidermis."

. . . . . "*Diagnosis.*—Every medical man knows well the occasional difficulty in determining whether certain cutaneous eruptions are truly scabious or not. This is the more annoying, as the appropriate treatment depends on the correct diagnosis of the disease: an error in this respect may be followed by the most troublesome consequences. Scabies may be confounded with some of the forms of *eczema.* In the latter disease, the vesicles, even when they are situated in the fingers and on the inside of the arms, are flattened on their summits, and not pointed as in the vesicles of genuine scabies; likewise, they are more congregated together, and they appear on the back of the hand as well as along the line of the flexure of the joints; the pruritus too accompanying *eczema* is more of a burning or scalding character, and not subject to those exacerbations so characteristic of the genuine itch.

*Herpes* appears in patches, and can scarcely be confounded with *scabies.*

*Prurigo* is not so readily distinguished. Apart however from the primitive characters of the eruption, it in most cases affects chiefly the back, shoulders, and the limbs, rather along the line of extension, than along that of flexion.

The papulæ exhibit at their apices a blackish crust; and the itching, though troublesome, is never comparable to that of genuine scabies.

But we must acknowledge that, in some cases, it is impossible to discriminate with confidence the exact nature of the eruption."

. . . . . "It is not unfrequent to observe in persons, who have been affected with scabies, a vesicular eruption returning every year, especially during warm weather. This affection is not strictly scabious, nor is it contagious. It does not therefore require the specific treatment necessary in itch; and very generally it will disappear under the local application of cooling washes."

. . . . . . "*Treatment.*—The ointment generally used by M. *Biett* is one of the best. It consists of two parts of sulphur, one of subcarbonate of potash, and eight of lard. The lotion of *Dupuytren*—composed of four ounces of sulphuret of potash, a pound and a half of water, and half an ounce of sulphuric acid—will often succeed where the patient is unwilling to rub in the sulphur ointment. It has however the twofold disadvantage of being very irritating, and very offensive to the smell.

One or two brisk doses of a cooling purgative will always be useful. No other internal medication is necessary."—*La Lançette Française.*

---

### On Convulsions during Pregnancy and Delivery.

The following general conclusions close a very able memoir on the above subject from the pen of one of the most experienced accoucheurs in Paris, M. *Capuron.*

1. Convulsions occur much more frequently during a delivery at the full period than during a miscarriage—doubtless, from the greater severity of the pains and the consequent greater disturbance of the circulatory and nervous systems. Indeed it is truly astonishing that such protracted suffering as almost always accompanies a first labour does not in every instance induce some convulsive attack.

2. The majority of the women, who are seized with convulsions during pregnancy or labour, are of a sanguineous and plethoric constitution, and usually of an irritable and highly nervous temperament.

3. The attack is often preceded by some precursory symptoms, such as headache, confusion, noises in the ears, twitches of the tendons of the fingers or toes, or of the muscles of the face, and a tendency to bewilderment and forgetfulness. The patient is usually much depressed in her spirits, and very apprehensive of the result of her labour.

Perhaps, however, generally the convulsions come on unexpectedly and without any premonitions.

4. The convulsions, after lasting for a longer or shorter period of time, usually terminate in deep somnolence, during which the respiration is heavy and more or less stertorous, and the pulse is full and large, such as is commonly felt in sanguineous apoplexy: occasionally a partial tetanic contraction of the jaws continues for a considerable time after the abatement of the general spasms.

5. From what I have observed, I am inclined to be of opinion that an attack of convulsions during a premature labour is on the whole more dangerous than a similar attack if the labour should be at the full period of gestation.

We might expect that this should be the case, when we consider that the cervix uteri is generally harder, less pliant, and more resisting, if labour happens to come on in the seventh or eighth month of pregnancy.

6. According to the results of my experience, local and general bloodletting and the use of warm relaxing baths are the most powerful means both to prevent and to arrest the attacks of puerperal convulsions. The blood-letting relieves the congested vessels of the head, and probably also the sanguineous accumulation in the uterus, and the warm-bath takes off the spasmodic state of this organ and of every other part of the body, by inducing a derivative action towards the surface. As auxiliary means of occasional utility, the extract of belladonna rubbed on the cervix uteri, and some of the milder preparations of opium given internally, may be mentioned with praise.*

7. If we should find on examination that the cervix uteri forms a rigid band around the head or neck of the child, and that the labour-pains make little or no impression upon it, even after blood-letting and other relaxing means have been used, we should not hesitate to divide the constricting portion at one or two places of its circumference with a bistoury. In all such cases, it becomes the accoucheur to ascertain the state of the urinary bladder; as it has been found, in more than one instance, that over-distention of this viscus has powerfully predisposed to, if it has not actually caused, the occurrence of convulsions during accouchement.

If the head of the child be within reach of the forceps, we should never hesitate at once to finish the labour by extraction. But if this be impracticable, and the convulsions still continue, recourse must be had without delay to the use of the perforator and crotchet.—*L'Experience.*

### Chilblains treated with Mustard Baths.

Mustard powder enters into the composition of several ointments which, at different times, have been strongly recommended in the treatment of chilblains: it is contained in the pommade of *Swediaur*, which has been so long known and esteemed.

A writer in the *Journal des Connoissances Medicales* alludes to the good effects of mustard baths in the following terms:—

"A friend of mine suffered so much from chilblains, that he could scarcely walk across his room. As he was afflicted with severe headaches at the same time, I advised him to use a sinapised foot-bath for two or three nights. Finding himself much relieved both in head and feet, he continued their use for nearly three weeks. This was in the month of November. He had no return of his chilblains during the whole of the winter; and, in the following year, by having recourse to the baths, he again remained entirely free from his old most troublesome complaint."

*Remark.*—A very good application to that most vexatious malady, a chilblain, is the diluted tincture of iodine. The effects of this remedy resemble, in many respects, those of a solution of nitrate of silver; both having a very marked, and often a singularly beneficial, influence on cutaneous complaints, especially when these are connected with a chronic or subacute inflammation of the part. We have recently used the iodine tincture with good effects in some forms of porrigo.—(*Rev.*)

### Memoranda on Cutaneous Transplantations.

. . . . . . . . . . "When erysipelas comes on in the face after a rhino-plastic

* M. *Capuron* omits to mention two of the most powerful remedies in the subjugation of puerperal convulsions—viz. the tartrate of antimony, and full doses of camphor. The former may be given in doses of half or of a whole grain, combined with a drachm of syrup of poppies, every quarter of an hour, (after venesection) until the spasm relaxes. From five to ten grains of camphor with fifteen to twenty drops of tincture of henbane, administered every half hour or so, constitute also a most potent remedy.—(*Rev.*)

operation, it is usually arrested at the edges or limits of the restored part. This was especially remarkable in the case of a patient operated on by *Dieffenbach,* at the hospital St. Louis in Paris.

M. *Ricord* also has published a similar instance. On the seventh day after the operation, the patient began to experience pain under the chin, and at this part an erysipelatous redness was visible. Next day this had extended over the entire face, the newly-made nose alone remaining intact; it was cold, insensible, and, as it were, indifferent to what was going on all around it. Although the local inflammation was very severe, it was ultimately subdued; yet at no time did the new nose participate in it.

*Dieffenbach* mentions that one of his patients, on whom he had operated, was attacked with jaundice, during which the entire face—with the exception of the nose, which remained white—became as yellow as a guinea."

M. *Phillips* insists very particularly on the necessity of keeping the newly-formed organ very cool with refrigerant lotions, and even of drawing blood freely from it as well as from the adjacent parts, and never of applying—as has too often been done—spirituous or other stimulating washes for the purpose of exciting the circulation. The condition of the transplanted tissues seems to be very nearly analogous with that of frost-bitten parts; in the case of which, it is well known, the application of heat and exciting substances must be carefully avoided. As illustrative of the same fact, M. *Phillips* goes on to state :—

. . . . . . . . " *Dieffenbach* remarked that in cholera patients, who recovered, the healing of wounds was effected with greater rapidity than in persons in perfect health; as if the diminished activity of the cutaneous circulation rendered it better fitted for any plastic processes. He observed that the wound bled very little, if at all, the dermis being almost void of blood; and that the edges of the incision were not red as usual, but rather of a yellowish-red colour. . . . . . . Again, if we call to mind the surprising unions of parts which have been entirely separated from the body, are we not led to conclude that the immediate union of wounds will be the more readily effected just in proportion as the parts are the least gorged with blood? The management, therefore, of rhino-plastic injuries should be so directed as to bring and keep the tissues in this state of vacuity."

M. *Phillips* proceeds to point out an error which is too commonly committed by most surgeons in the treatment of recent wounds, that of bringing the edges in contact as quickly as possible after their division, and before the oozing of blood has ceased. Now, it is not until this has quite stopped, that the secretion of the coagulable or plastic lymph commences; and it must surely be obvious that, if any coagula of blood are interposed between the lips of the wound, the agglutinative process must necessarily be impeded. *Dieffenbach* observed, in some experiments which he performed on rabbits, that the chances of union, after the transplantation of a portion of the integuments, were always greater when the flap was not replaced for several minutes after it had been excised.

In most of the cases, too, of successful union of detached noses or fingers in the human subject, some time has accidentally elapsed between the occurrence of the accident, and the re-application of the part."

. . . . . " That a vitality continues for a considerable time in parts detached from the body, is often very apparent after some operations.

Thus in a case of hydro-sarcocele, where *Dieffenbach* removed a large portion of the skin along with the diseased mass, he noticed that, in about a quarter of an hour afterwards, this portion of skin had contracted upon itself, so as to exhibit the usual puckered appearance of the scrotum on leaving a very cold bath. On stretching it out, and pouring cold water upon it, it again contracted into numerous wrinkles."

M. *Phillips* proceeds to state that—

" When a part is entirely detached, its natural red colour disappears, and it becomes very pale, although very little blood has flowed from it. The vessels of the surface contract, and drive the blood into the sub-dermoid tissues. In a few minutes after the separation, this great paleness diminishes; some dark blood oozes from the divided edges; and we may ascertain with a magnifying glass that it is expelled by the contraction of the vessels. If the blood be wiped away, the wound again becomes red; but the blood is now thinner, and if again removed it becomes quite watery, and the edges begin to be coated with coagulable lymph; they then contract upon themselves, so as to cause the centre to bulge out." *

Should the hæmorrhage from any of the divided blood-vessels, after auto-plastic operations, prove troublesome, *torsion*, and never the *ligature*, should invariably be had recourse to. The presence of a ligature, however minute, is always most unfavourable to immediate union. If the stopped blood-vessel seems gorged with blood after the hæmorrhage has ceased, one or two scarifications should be made, to remove the sanguineous congestion; which we have already explained is very prejudicial to the success of the reparative process.

M. *Phillips* thus points out some of the anatomical conditions of the flap taken from the forehead in a rhino-plastic operation, on which its success very materially depends.

" It is indispensable that it should contain one artery at least. Hence the importance of prolonging the incisions to the angle of the eye, so that the pedicle of the flap contains the internal angular artery to supply it with blood. And then again, it is equally necessary that the blood, which is conveyed to it by the arteries, should be returned by veins, else the new nose will perish from congestive asphyxia. Indeed it is wonderful that this does not occur oftener than it really does, when we consider that not only are almost all the direct communicating branches between the arteries and the veins cut across, but that the blood in the divided arteries is prevented, in a great measure, from escaping, in consequence of the compression of the edges of the flap by the numerous sutures used to retain it in position."

" The destruction of the flap may arise from three different causes—from defective circulation, from excessive or superabundant circulation, and from suppuration.

If the pedicle be twisted too firmly, the vessels must necessarily be compressed, and the circulation through them be consequently more or less interrupted: the flap in consequence becomes pale, loses its heat, and speedily mortifies. But this does not occur so frequently as the mortification from accumulation or congestion of blood in consequence of its obstructed return through the veins. Under such circumstances, the mortification usually takes place in that part of the flap which is furthest distant from the pedicle.

---

* The convexity of the flap exists even after the most successful union; it is the more decided when the substance of the flap is thin, and much less so when a layer of muscular substance has been detached at the same time.

This tendency of the flap to contract around its edges, so as to cause the centre to become convex, is of great utility in rhino-plastic operations; as it is by this invariable law that the surgeon is able to secure a proper prominence to the new nose. When a flap has a free edge, which is not brought and kept in contact within another incised surface, this edge turns inwards upon itself and contracts very close adhesions. It is in this manner that the nostrils of the new organ are apt to become quite obliterated, if precaution has not been taken to evert and secure by a stitch or two a portion of the flap, so as to induce an immediate union.

After the asphyxia now described, suppuration is the most formidable evil to be dreaded in the management of rhino-plastic operations. The destruction commences at the edges, which swell, puff out, and separate from each other. This calamity is more apt to occur when the twisted suture has been used."*—*Bulletin Medical Belge.*

### M. Trousseau on the Pommade Ammoniacale, and on Heat as Vesicants.

The formula in the French Codex for the preparation of *Gondret's* Ammoniacal Pommade is as follows:—Take of mutton suet one ounce, lard one ounce, and liquid ammonia (of 25° strength) two ounces. Melt the suet and lard in a wide-mouthed bottle, and then, having added the ammonia, stopper it and shake the mixture well together. Keep the bottle closed in cold water—shaking it occasionally—until the pommade has become quite cold.

M. *Trousseau* observes, that the pommade made according to these directions, is generally much too hard and consistent for use, at least unless the weather be warm. He proposes two different formulæ, one for Summer and the other for Winter. The former is to use three parts of lard, one of suet, and four of ammonia; and the latter to use equal parts of lard and of the ammonia.

The *pommade,* which is met with in most druggists' shops in Paris, is far too weak, in consequence of its having been prepared with ammonia of insufficient strength, and probably also from its being kept too long.

There may be another reason still: if the ammonia be added while the fatty matters are very hot, a great portion of it must be volatilised, and the pommade is necessarily weakened.

In applying this vesicatory, some recommend that a portion be put into a thimble, or in a short glass tube, and held firmly on the skin for some time, to prevent the escape of the ammoniacal vapour; while others advise, that it be spread directly on the part which is then covered with a small portion of diachylon plaster.

M. *Trousseau* disapproves of both methods, as "it is indispensable," he says, "that the effects of the application should be visible all the while." When first applied, the patient experiences a sense of coldness in the part; then a minute or two afterwards a slight burning heat, which gradually increases for three or four minutes, at which time it is at its maximum: it then remains stationary, and sometimes even begins to abate, although the *escharification* of the skin is going on all the time.

We must not, therefore, judge of the effects of the ammonia solely by the effects it produces.

The rule given by M. *Trousseau,* for regulating the length of time for its application, is to watch the appearance of the red circle around the part: whenever this is observed, the pommade should be removed. It is sometimes even not necessary or proper to wait until the red areola be observed, as the epidermis may be raised, before it appears.

The length of time necessary for the pommade producing its full effects,

* If the destruction from this cause be considerable, it may be necessary to perform a second operation, and re-transplant a fresh portion of healthy integument. But if it be only at one angle, we may succeed by the repeated application of the tincture of cantharides, in exciting the granulating process sufficiently to fill up the ulcerated space.

varies much according to the part on which it is applied: sometimes it will vesicate in two minutes, at other times ten or twelve minutes are required.

As the inflammatory areola does not always make its appearance, M. *Trousseau* recommends that the pommade be wiped off at the end of five or six minutes, and reapplied if necessary; this may be repeated until the epidermis begins to be raised. We are never to continue the application until any appearance of a vesicle or blister is observed; but only until we find that the epidermis can be detached by rubbing it gently with the finger. We frequently find that it can then be removed in one piece, and that the chorion beneath can be fairly exposed. *Now this is the very condition that is desired.* The dermis is not escharified in any degree; but continues to retain its absorbing energies, so that we may depend upon the success of any endermic medication that we may wish to try.

Should the pommade have been so long applied, that a complete bulla has been raised, it will generally be found that the dermis, from having been too much irritated, does not absorb very readily; and, moreover, that a cicatrix or mark is left after the blister has been healed. This latter consideration is not to be neglected, seeing that the pommade is so extensively used in the treatment of facial neuralgia in both sexes.

### *Heat as a Vesicant.*

We shall only mention the application of boiling water under this head. By far the most convenient method of using it is in the manner suggested by M. *Mayor,* and known in France under the somewhat droll appellation of M. *Mayor's hammer.* It consists in simply immersing the iron part of a common hammer in water boiling on the fire for a minute or two, and then applying its round disk on the part which we wish to vesicate. The pain is sharp, and when the hammer is removed, the skin is observed to be discoloured and as it were somewhat sunk in. M. *Trousseau* very justly remarks, that the great objection to the use of heat, applied in any way, for the purpose of inducing vesication, is that the part is very generally more or less completely escharified—in consequence, perhaps, of the albuminous portion of the animal fluids being necessarily more or less completely coagulated. He approves of M. *Mayor's hammer* as a very good means of producing an eschar, such as may be desired in the neighbourhood of diseased joints; but rejects it altogether as a vesicant in cases where we wish to try the endermic effects of remedies in neuralgiæ and such like diseases.—*Journal des Connoissances Medico-Chirurgicales.*

---

### ON THE GREY DEPOSIT UPON URINARY CALCULI, BY M. CIVIALE.

The main object of M. *Civiale* in the following remarks (derived from the third volume of his recent work on Calculous Diseases) is to shew that the use of chemical remedies is not only quite inefficient, but is positively pernicious, in a multitude of cases, by promoting the deposit of additional matter on the calculi which are in the bladder.

After alluding to the great variety, in point of chemical composition, of calculi, he says:—

" We cannot ever know exactly in the living subject the species of stone which exists in the bladder; it is not possible to do more than acquire merely vague notions, which are quite insufficient to direct us in the choice of our remedies.

(This assertion is far too positive and dogmatic: the English surgeon knows very well that he can generally predict the nature of the calculus, before it is extracted.—(*Rev.*)

Hence there must happen one of two things; either the remedies employed are inefficient, or they must exert some real effect, which may be useful in one case, but injurious in another. In my opinion, there is not a single decisive instance on record of the solution of a calculus in the bladder. Alas! there are, on the other hand, too many in which the disease has been aggravated by the use of chemical solvents. If the use of these remedies does not tend directly and immediately to promote the deposit of further matter on the surface of the existing calculus, it is, in a multitude of cases, apt to induce various morbid conditions of the urinary apparatus, under the influence of which the grey or white layer, of which I am about to speak, is generally secreted. No one, indeed, who is in the habit of seeing much of calculous diseases, can fail to be struck with the frequency of this layer or calculi, when alkaline remedies have been taken for a length of time."

M. *Civiale,* like so many of his countrymen, is apt to carry all his doctrines *a l'outrance.* The *abuse* of alkaline remedies has, we are ready to admit, often done harm; indeed it must do so on chemical principles. The rule of practice, which is invariably followed by the scientific surgeon, is every now and then to *test* the urine of a calculous patient, and never to continue the employment of either alkalies or acids beyond the neutralization of the acid or alkaline condition of the secretion. It is therefore scarcely necessary for M. *Civiale* to condemn the use of alkalies in the phosphatic or oxalate of lime diathesis, or to take any credit to himself for having pointed out the evils of such a practice: no one in the present day is so foolish as to regard alkaline remedies as universal solvents of calculi.

M. *Civiale,* indeed, mentions the names of *Brodie* and *Prout,* as men who have paid attention to the occasional alkaline state of the urine, and the circumstances which are apt to induce it; but his notice of these distinguished practitioners goes no further; and he takes the sole credit to himself for having showed that a diseased state of the bladder and kidneys has a marked influence on the nature of the deposit from the urine. We do not mean to rob M. *Civiale* of his share of the credit of having improved the treatment in calculous diseases; but let him be satisfied with his own portion.

In illustration of his particular views he makes the following remarks on a case, where it had been alleged that the mineral waters of *Vichy* had begun to exert a solvent effect on a urate of soda vesical calculus, in consequence of its surface exhibiting a layer of white deposit:—

"Now this white layer, which our confrere imagined to be the effect of the mineral waters, was solely the result of a new deposit, which had taken place in consequence of the inflammatory action on the mucous coat of the bladder. Whatever be the nature of the calculus, no sooner is a vesical catarrh induced, than this white or greyish deposit inevitably supervenes, whether alkaline or acid remedies be taken or not. I could quote a thousand cases in proof of this point: let one suffice. . . . . In an old gentleman, the bladder was the seat of a purulent catarrh, which ultimately proved fatal. Twenty-six minute calculi—all of uric acid or urate of ammonia—were found, on dissection, in it; these were all invested with this ash-coloured deposit. The left kidney contained so large a number of red granules of uric acid that it took a considerable time to count their number. But they were all of a lively red colour, and not one of them exhibited the greyish surface observed on the vesical calculi.

Surely then we must admit that this greyish deposit was the result of the vesical catarrh, as all the calculi, vesical as well as renal, were essentially and primarily of the same nature."

M. *Civiale* next alludes, in illustration of his views, to the deposit which is apt to take place on the extremity of a sound or catheter, when left so long in the bladder as to give rise to irritation of its mucous membrane: this being always of the greyish character, such as we observe on the surface of calculi.

The quickness with which this takes place is always in proportion to the amount of the irritation excited, and to the degree of morbid action induced in the mucous surface of the bladder. To prove that such is the real cause we have only to omit the use of the sound for some time, and use means to soothe the irritation ; and it will be found that, whenever this ceases, the tendency to deposit is at once checked.

The relief, which patients derive from the use of alkaline waters, is in reality often very delusive. The urine indeed, which has perhaps been turbid, may have become limpid and unirritating, and, with this change, the calculous symptoms may be considerably mitigated. Even small calculi, which have lodged for a considerable time in the bladder, may be now discharged ; and yet, all this while, the larger calculi, which cannot pass through the urethra, may actually have increased in size.

The explanation of these seemingly contradictory occurrences is not so difficult as may be imagined. The diuresis induced by the use of the alkalies will account for the change in the appearance of the urine ; and as to the relief of the calculous symptoms at the same time, this is very often owing to the sharp edges of the calculi becoming rounded from the deposit of the white layer upon them. M. *Civiale* is decidedly of opinion that in numerous cases the prolonged use of alkalis and alkaline waters, such as those of Vichy, Carlsbad, &c. has the effect of inducing irritation of the vesical lining, and of consequently favouring, instead of checking, the further deposition of earthy matter on the surface of calculi in the bladder.

The relief in such cases is altogether attributable to the surface of the calculus becoming smoother and its edges more rounded, and not to any incipient solution of its substance, as is generally imagined. That such is the truth, we have often a most satisfactory proof in the treatment of calculi by lithotrity.

The fragments of a calculus, if they lodge in the bladder for some time after it has been broken, and especially if their presence has excited irritation of its mucous membrane, are found to acquire gradually a more rounded figure, in consequence of the sharp angles becoming invested with a coating of the grey-coloured deposit. There has been no wearing down of the angles, but on the contrary, an addition of substance to and around them. That such is the case may be easily shewn by breaking the white crust on their surface ; and then all the sharp angles are at once perceived.

Because this white crust often exhibits a roughened, and as it were corroded, surface, it has been hastily imagined that the alkalies, which have been administered, have been exerting a solvent action on the crust, whereas, in truth, a continued deposition of new matter has actually been taking place all the time. That small calculi are occasionally discharged by the urethra, after a prolonged use of alkaline remedies, is quite true ; but such an occurrence is generally attributable not to the diminished size of the calculi in consequence of partial solution, but to the rounded form, which they have acquired, causing less irritation and spasm of the urinary passages.

M. *Civiale* has, in his fifth letter, adduced many cases in proof of the preceding views.

In particular, he mentions that of Dr. *Bigel*, a physician at Warsaw, who, after undergoing the operation of lithotrity, went to Carlsbad for the purpose of drinking its celebrated waters. While residing there in 1835, he passed a number of minute calculi of a white appearance and not very firm consistence, whereas immediately and for some time after the operation the fragments, which were discharged, were all hard and of a brown colour.

Dr. *Bigel* was so delighted with this *seemingly* most favourable change, that he published a memoir on the remarkable solvent powers of the Carlsbad waters. He there stated that the calculi in his own case were white, not only on the surface, but nearly throughout their entire thickness. When however they were examined by M. *Creusburg*, a chemist, a very different report was made; he found the exterior white crust was very thin and might be readily detached with the nail, and that it enveloped a hard red kernel, so to speak, which required the blow of a hammer to break it in pieces. The calculi were of different kinds; some were of reddish-grey exterior, others of a brownish red, and others were nearly white. The first set consisted of uric acid, urate of ammonia, oxalate of lime, and of a minute portion of oxide of iron; while in the white-coloured ones the nucleus consisted of the same ingredients, now enumerated, but the outer crust or envelope was composed of phosphate of lime with a minute proportion of phosphate of soda, urate of ammonia, and mucus. "All these results," says M. *Civiale*, "are highly important; more especially the last-named one; as we may fairly infer from it that no particular action had been going on in the bladder of Dr. *Bigel* different from what occurs in calculous patients who are not taking any mineral waters."

The relief, which Dr. *Bigel* experienced whilst at Carlsbad, was far from being of long duration. Two months afterwards he began to experience all his former distress, and again he was obliged to have recourse to a lithotritic operation. For the next twelve-months he continued free from any urinary concretions, although, every now and then, he passed a quantity of gravelly matter. M. *Civiale*, adds:—

"Dr. *Bigel*, who does not seem latterly to believe in the solvent virtues of the Carlsbad waters, attributes the cessation of his gravel symptoms to the return of gouty rheumatism in his shoulder, to which he had been subject for nearly thirty years before his urinary complaint commenced, and which, he thinks, has been recalled by the use of the sulphureous waters of Warmbrun."

M. *Civiale*, after some further remarks on the indiscriminate and prolonged use of alkalis and alkaline mineral waters, informs us that one or two of the most experienced physicians in Paris have been led by his writings to entertain similar opinions to those now announced.

"MM. *Leroy* and *Segalas*, in 1837, spoke most favourably of the solvent effects of the mineral waters of Vichy; but since that time they have found reason to change their opinions. They have communicated to the Academy several facts, which tend to prove that, under the influence of these waters and also of alkaline preparations, calculi have increased in size."

In conclusion, M. *Civiale* repeats his opinion, that there is not one authenticated case on record of a solution of a urinary calculus in the bladder from the use of any remedies, and that, on the contrary, we have too much reason to suspect that, in a great number of instances, the long-continued administration of pretended solvents has seriously contributed to the aggravation of the evil.—*La Lançette Française.*

---

### On the Ophthalmia in the Belgian Army.

M. *Caffe* was appointed in 1838 by the minister of commerce to visit Belgium, Holland, and Prussia, with the view of examining, on the spot, the epidemic ophthalmia which for many years past has affected the troops of the Belgian army. This ophthalmia was first noticed, at least as an epidemic, in 1814; but it has been more particularly since 1830, after the movements of the last revolution, that it has prevailed with great severity, and to such an extent that it has usually attacked at least one-eighth of the entire army, and in some regiments nearly one-half of the soldiers. Upwards of a hundred thousand persons in Belgium have suffered from it since its first invasion; and an immense number of these have in consequence totally lost their sight.

The Belgian Government appointed a permanent committee, composed of their most distinguished surgeons and physicians, and authorised them to invite, at the national expense, some of the eminent oculists of Vienna and Berlin to investigate the cause of this wide-spread pestilence, and to consult as to the best means of arresting it. But it would seem that not much success has resulted from their enquiries; for in 1838 there were still 5000 ophthalmic patients in an army of not more than 50,000 men. A great number of the soldiers were blind, and there were no signs of a cessation of the epidemic.

The characteristic symptoms of this ophthalmia may be briefly described to be an extreme vascularity, tumefaction and softening of the conjunctiva, the development of granulations on the folds of this membrane, and the secretion of a fluid, at first serous, then muco-purulent, and lastly entirely purulent. When the disease is severe, the tumefied eyelids become of a purplish red colour, the purulent discharge is immense, a pulpy whitish spot is observed at one point of the cornea, which at length gives way, either from ulceration or gangrene; and then the humours of the eyeball escape and the sight is irrecoverably lost. In less severe cases the entire thickness of the cornea is not affected; but even then the sight is perhaps left almost wholly interrupted, in consequence of an extensive and permanent opacity. In other cases again—and these seem to be most common at present—the ophthalmia is chronic from its commencement, or becomes so after a very short acute stage; the conjunctiva remains injected and swollen, the granulations continue, the discharge is less copious and of a muco-serous character, and the cornea becomes nebulous: this state may last for a considerable length of time.

M. *Caffe* is himself quite satisfied that the Belgian ophthalmia is truly contagious, and communicable from one person to another, not only by the direct application of the discharge, but also through the medium of the atmosphere by some infectious miasm.

Indeed no one can possibly doubt that the disease may be communicated by the inoculation of the purulent matter on a healthy eye: the proofs are too obvious and decisive. But it is certainly more difficult to establish the miasmatic infectiousness of the disease; M. *Caffe* however feels equally satisfied upon this point also.

The contagious character of the epidemic does not exclude the idea of its spontaneous development; although, it must be confessed, the actual cause or causes of the disease have hitherto escaped detection. Some writers indeed have imagined that it has been imported from Prussia or Egypt; but this seems quite fanciful.

It is worthy of remark that the history of the *blennophthalmia*, which exists almost permanently in the Foundling Hospital and in the Orphan Asylum at Paris, confirms in almost every point the opinions of M. *Caffe* on the nature of the Belgian ophthalmia; there being a very striking resemblance between these two forms of the disease.

M. *Caffe* had in 1832 observed a similar epidemic at the temporary hospital of Bons-hommes, where upwards of a hundred children were crowded together during the prevalence of the cholera;—a purulent ophthalmia made its appearance among them, and several of the nurses and one or two of the medical attendants caught the disease. There had been no ophthalmia in the hospital before.

As to the *treatment* of the Belgian ophthalmia, it is admitted by all that the severity of the disease is aggravated by the crowding together of many patients in the same ward, and decidedly mitigated by dispersing them. M. *Caffe* advises that, when the disease makes its appearance in a regiment, the soldiers ought to be bivouacked in the open air, provided the weather be sufficiently warm and dry. It is at least quite necessary to separate at once every man who is even threatened, far less already affected, with this disease. If this practice were more rigidly executed, M. *Caffe* is of opinion that the epidemic might be arrested. He had seen almost every remedy tried; he was sorry to confess that no line of treatment seemed to have that influence in arresting the disease, which might warrant him in giving it a decided preference.

*M. Baudelocque,* in commenting on the observations of M. *Caffe,* and other preceding speakers, mentioned that, on more than one occasion, he had observed at the Hôpital des Enfans an epidemic purulent ophthalmia seemingly arrested by thinning the number of the children in the wards, and dispersing them in separate rooms. Until this was done, every remedy that was used proved inefficient; but afterwards it was observed to be comparatively easy to treat the disease. It is doubtful indeed whether the purulent ophthalmia in infants and children be decidedly contagious; but at all events it is wise to separate the diseased from the healthy, and keep the latter quite apart. No remedy is on the whole so efficacious as the local application of a solution of the nitrate of silver, not omitting at the same time the application of one or more leeches to the temples, of a blister to the neck, and the internal administration of appropriate medicines.

M. *Velpeau* confessed that he had not seen the Belgian ophthalmia, but he was strongly impressed with the idea that the same mode of treatment, which was so successful in his practice in the other forms of purulent ophthalmia, would, if rightly pursued, be found most useful in the cure of the disease. He alluded to the use of a strong solution of the nitrate of silver—from half a drachm to a whole drachm of the salt dissolved in an ounce of water. As a matter of course, the employment of other means, constitutional as well as topical, is not to be neglected.—*Gazette Medicale.*

---

### On the Purulent Ophthalmia of Children.

The following remarks apply to that form of purulent ophthalmia which usually occurs epidemically among children of three or four years of age—a form of the disease which is very different from that which is often observed in infants soon after birth, and which, it is believed, arises from the contact of the acrid mucus of the vagina with the eyes of the infant during the process of delivery.

The former is of frequent occurrence in Paris, and is usually observed during the early months of the year. It is observed not only to attack a number of children at the same time, but also, especially in a hospital, to propagate itself from one patient to another, who may happen to be next to him—thus seeming to be contagious as well as epidemic.

It is one of the most alarming diseases of childhood, in consequence of the extreme rapidity of its progress, and the tendency there is to perforation and consequent destruction of the eye, if the symptoms are not promptly subdued. Occasionally it proves fatal; but such a termination is generally, we believe, attributable rather to the improper treatment which may have been pursued than to the mere severity of the disease itself—unless indeed the child is in an unhealthy state at the time of its seizure.

The following two cases may be aptly quoted here in illustration of this remark.

*Case* 1.—A child, four years of age, had been suffering with cough and purging for several days before the right eyelid was observed to have become of a purplish-red colour, and to be swollen and quite closed: a purulent matter also kept oozing out in considerable quantity. Leeches were applied to the temples, and emollient washes to the eye. Four days afterwards, the cornea was perceived to be much softened, and to threaten immediate perforation. The left eyelid also had now become affected in nearly the same manner as the right one had been four days before.

The same line of treatment was unfortunately continued; and both eyes were irretrievably lost.

The constitutional symptoms did not, as is usually the case, abate; and the patient died on the 15th day of the ophthalmia.

*Case* 2.—Two days before the death of the former child, another child in the same hospital, (Hôpital des Enfans,) and who slept in the adjoining bed, exhibited the early symptoms of a similar ophthalmia. The system at this early period was not much affected. Leeches and emollient applications were used. Ultimately both eyes were destroyed from rupture of the cornea, and the escape of the contents of the balls. In this case, however, the constitutional symptoms abated after this unfortunate event, and the young patient gradually recovered.

The mode of commencement of this formidable disease is usually insidious: there being little or no fever present at first, and the eyelids only seeming to be the parts affected. These acquire a deep-red or purplish hue, and become puffy, swollen, and closed. There soon follows a copious oozing out of a thick mucous matter, which seems to be secreted almost as quickly as it is removed, and there is also an abundant flow of tears, whenever the lids are separated. Still there is neither much pain nor heat in the parts, until the second or third day, when the eyeball itself usually becomes involved, (if the disease has not been arrested,) and the constitutional symptoms are aggravated. Soon after this period, in unfortunate cases, the cornea bursts, the contents of the eyeball escape, and the eye is then irretrievably gone.

It is rare that both eyes are affected from the first at the same time; although it is very common to observe one eye to be attacked with the disease in a few days after the other.

As we have already remarked, the destructive effects of this formidable disease are, in not a few instances at least, referrible to the erroneous line of treatment which is too frequently pursued. It is not by the use of antiphlogistic and emollient remedies that we can hope to arrest or counteract its rapid and disorganizing progress. Indeed whatever has the effect of debilitating the system seems to favour, rather than to arrest, the tendency to rupture of the cornea and the consequent destruction of the eye.

Instead therefore of bleeding and other powerfully depressing measures, the employment of gastric evacuants, and especially of emetics, is greatly to be preferred. The action of vomiting is not merely to cleanse the stomach and primæ viæ of all offending matters, but also to cause a powerfully derivative action from any suffering organ to the skin, and thus determine an equable flow of blood to the entire surface of the body.

After the free operation of an emetic, which should be repeated at short intervals in some cases, a blister should be applied to the nape of the neck or to one of the arms, with the view of diverting the incipient morbid action from the eyes. A large dose of calomel may also at the same time be given; and this should be repeated at short intervals, either alone or in combination with jalap or some other purgative, so as to cause copious evacuations from the bowels. When once this is effected, the diet allowed may be generous or even stimulating, and a moderate quantity of wine may also be allowed.

As to the local treatment of the purulent ophthalmia of children, we have already observed that the use of antiphlogistic and relaxing remedies is decidedly hurtful. Not but that if symptoms, local or general, of active inflammatory action be present in a case, as occasionally happens, it may be necessary to have recourse to smart antiphlogistic measures; but such a case is of exceptional and not of frequent occurrence. But under no circumstances is it prudent to continue the use of such a practice for any length of time; all that is requisite is to remove the active symptoms; and when this is effected, no time should be lost in resorting to some powerful topical application directly to the eye. Perhaps on the whole the most useful of all is a strong solution of the nitrate of silver, ʒss.—ʒj. to an ounce of rose water, of which a few drops are to be introduced between the lids, three or four times a day. Some surgeons recommend highly a solution of one grain of corrosive sublimate in eight ounces of distilled water as an eye-wash to be frequently applied; while others prefer a strong opiate collyrium, prepared by dissolving two scruples or a drachm of opium in a pint of tepid water. But none is on the whole so generally useful and so decidedly potent as the solution of the nitrate of silver. By the judicious use of this means, aided with the employment of calomel and emetics and the application of one or two blisters to the neck, a cure will often be effected in two, three, or four days.—*Bulletin General de Therapeutique.*

---

### M. Lisfranc on certain Fallacies in Surgical Works.

From a lecture recently delivered by this celebrated surgeon at the Hôpital de la Pitié, the following extracts on some of the more common errors which are found in surgical books are worth perusal.

*Facility of Diagnosis in general.*

"Whatever may be told you in other places to the contrary, I can positively assure you that the diagnosis of diseases is by no means, generally speaking, so very difficult. Lecturers and writers are too apt to frighten you by representing it as encompassed with difficulties which are only to be overcome with much study and erudition. Do not believe such statements; diagnosis is on the whole tolerably easy; the nurses and other attendants in our hospital, although ignorant of scientific particulars, generally form a very shrewd guess of the malady with which each patient on their admission is affected, and can tell you when you arrive to examine them: it is rare for their judgement to be wrong."

This is all very true; but M. *Lisfranc* knows very well that nurses may indeed be clever guessers in a rough way, but that it would not be very safe to found any line of treatment on their diagnosis. We must not however deny that an experienced nurse is often more likely to be right than a mere *book-doctor*:—practice is every thing.

*Difficulty of Diagnosis in certain Cases.*

"Nevertheless there are cases, in which the obscurity of the diagnosis is so great that it is almost impossible to dissipate it. Yet if you open some works, you are told that every thing is simple. For example; Is there a chronic swelling of the testicle? they will tell you that it is quite easy to determine whether the swelling be of a scrofulous, scirrhous, syphilitic, or tubercular nature. But when you come to study the reality in the hospital, you then discover the fallacy of these scientific romances, written in the silence of the study and away from the spectacle of suffering humanity. You are astonished not to find that multitude of varieties or species of a disease which are described, and with great minuteness too, in the standard works of the day. Thus we read of 30 or 40 different varieties of ophthalmia, as if it was possible for any man to discriminate a sixth part of them. But to return for a few minutes to the subject of tumors, we may mention that no surgeon was more cautious in pronouncing on the nature of many morbid swellings than the late Baron *Dupuytren.* I remember a case in point: a patient had a large tumor situated at the inner angle of the right eye, and which alternately enlarged and diminished with the movements of respiration: it also became fuller by heat, and smaller by cold and by compression, recovering its original size whenever the pressure was removed. The case had been seen by a multitude of surgeons, who all gave some opinion or other; the general impression was, that the tumor was of an erectile tissue. *Dupuytren* alone confessed that 'he could make nothing of it.' An operation was performed; and it was then discovered that the swelling was caused by a large varix of the nasal vein resting upon a fatty tumour. Let me mention another instance where the diagnosis was very obscure and difficult.

A lady had suffered for twelve or fourteen years from pain and distress at the posterior part of the pelvis. Within the last few months a swelling had made its appearance there. She had been treated at different times for neuralgia, rheumatism, inflammation of the periosteum, &c. &c. At length she came to Paris to consult the leading surgeons of the metropolis. Some decided that there was a chronic abscess, connected perhaps with disease of the bone. An explorative puncture was made; and no sooner was the trocar withdrawn than a stream of blood sprung out with such force as almost to reach the ceiling of the room. Immediately after the operation, distinct pulsatory movements were felt, and a blowing sound was audible over the tumor, although previously there had not been a trace of either symptom.

These two cases will be sufficient to shew you how necessary it is to be cautious as to your diagnosis in certain obscure cases."—*Gazette Medicale.*

---

### ON MEDICAL METEOROLOGY.

It is a principle of medical philosophy, founded upon the experience of successive centuries, that there exist certain relations of causality and dependence between the physical characters of the seasons and the nature of the prevalent and endemic diseases.

Each season having a peculiar and individual nature determines in the animal economy a peculiar set of movements, and leaves certain impressions, which are the more decided and the more durable just in proportion as its action is exercised with greater energy and for a greater length of time.

The succeeding season comes in its turn to impress on living bodies a different series of movements; and these again give way to another series when the year has advanced two or three months. This principle of concordance of the seasons with what have been called the smaller epidemics—a principle so rich in clinical applications, so useful in assisting the physician in the diagnosis and in tracing the causes of many diseases, and so instructive in enabling us to discover their genuine nature and the rational and successful method of their treatment—had been already well laid down and illustrated in the writings of Hippocrates. In his treatise on air, waters and places, we read the following passage.

" Astronomy itself contributes not a little, but a very great deal, to the healing art; for it is well known that, along with the seasons of the year, the human body undergoes remarkable changes."

At various times the truth of this principle has been recognised and enforced; at other times it has been quite overlooked and neglected: but this has been more especially the case during the last fifty years. Medical men have in all ages been apt to carry certain doctrines to excess; hence what has been praised and exalted in one age has been too often been despised and utterly rejected in another. So apt we are in avoiding one error to fall into the very opposite one. *Luther*, with his usual fire, has very happily expressed this tendency of the human mind by comparing it to a drunken man on horseback; when he is lifted up on one side, he is apt to fall off from the other.

In the manuscript work " On the Diseases of France in their relations with the seasons of the year, or the medical and meteorological history of France," which Dr. *Fuster* recently communicated to the Royal Academy, we find the first serious attempt that has made for a long time to follow out the Hippocratic doctrine.

" The physical qualities, and, at the head of these, the temperature of the atmosphere, play the first and the most important part in the pathogenic influence of the seasons. In considering the question of aerial temperature, it is necessary to distinguish the absolute heat as indicated by the thermometer from the sensible heat as experienced by our feelings; for the effect of the one is by no means always proportionate to that of the other. Various considerations, derived on the one hand from meteorology and on the other from physiology, will account for such differences. Thus, quite independently of any variation of the absolute temperature, the atmospheric electricity, its dryness or moisture, its calmness or its disturbance with winds, and the state of the cutaneous perspiration as well as that of the nervous system of the individual, will be found to modify exceedingly the sensible heat of the air. . . . . It is especially on the association of the temperature with other conditions, such as the climate, the soil and its productions, the winds and waters, the light, electricity and magnetism, that the prevailing diseases of the different seasons depend."

There is seldom however any abrupt transition from one season to another, but rather a gradual and often imperceptible fusion of the characters of each, so that it is scarcely possible to affirm when one ceases and the other begins; and hence the medical constitution, so to speak, of the atmosphere at the period of meeting partakes of the characters of both. There is thus produced a mixed or complex pathological condition which results from the concurrence of the action of the season which is closing and of the action of that which is commencing. The experienced and skilful physician will not neglect the influence of such agencies in the treatment of many diseases; and thus his practice is not a little modified at one time from what it is at another in the management of the same malady.

" In Spring,—the weather of which is usually characterised by atmospheric vicissitudes of all sorts, partaking of the cold of Winter at its commencement and of the heat of Summer at its close,—the prevailing diseases are catarrhal and inflammatory in the first period, and catarrhal and bilious in the second. The organs of respiration and of digestion are those which are chiefly affected.

In Summer the increase of temperature causes a predominance of bilious affections. Nevertheless, as the Summer weather in France is usually very variable, partaking more or less of the characters of Spring and of those of Autumn, the bilious complaints almost always exhibit the features of a phlogistic or of a mucous diathesis. The gastric, hepatic, and intestinal systems are more particularly affected.

In Autumn, the recurrence of atmospheric varieties brings back the tendency to catarrhal affections as in Spring. There is however this great difference, that in Spring the catarrhs are usually associated with inflammatory affections, whereas, in Autumn, they are associated with bilious complaints; the alimentary organs being those which are most apt to be disordered.

During Winter inflammatory diseases are the most prevalent, and these are usually combined with catarrhal and other mucous affections. The sanguiferous system and still more the mucous system of the whole organism are those which are most compromised during this season."

Besides the decided influence which the characters of the different seasons exert on the nature of the prevailing diseases, it cannot be doubted that even accidental or sporadic diseases are modified more or less according to the season during which they occur. Thus a pneumonia occurring during Summer is apt to exhibit the impress, so to speak, of a bilious constitution. The symptoms indicate such a modification, and the treatment requires to be managed accordingly.

"This influence of the meteorologico-medical constitution of the weather is appreciable even on external diseases, wounds, and operations. *Dessault* and *Bichat* paid much attention to this subject; and the rich collection of the memoirs of the old Academy of Surgery furnishes numerous examples of allusion to it."

But not only do the different seasons of the year exert a decided influence upon the general characters of prevailing diseases, but even the various periods of a single day appear to have a somewhat analogous effect. It was a favourite doctrine of Hippocrates, that there was a strict relation between the action of the diurnal revolution and that of the annual revolution of the sun on the production and the progress of diseases; *sicut in anno continentur periodi ægritudinum, eodem modo unâ die.* The morning was the analogue of the Spring, the noon was the analogue of Summer, the evening represented Autumn, and night corresponded with the Winter. The year was thus considered only a lengthened day, and each day a year excessively contracted. Many of our medical classics, who have belonged to the Hippocratic school of medicine, have confirmed and illustrated these views of the old Coan by numerous observations: we may mention the names of *Sydenham, Triller, Baillou, Ramazzini,* and *Huxham,* as among the most distinguished writers of this school. The most important of their conclusions are these:—

"Various diseases exhibit certain features of change during the day and during the night; patients too, in their turn, have their feelings affected differently in a similar manner; and physicians have remarked that, even at different periods of a single diurnal epoch—that is to say in the time between the rising and setting of the sun—diseases often exhibit diverse phenomena.

Thus inflammatory diseases, those which are characterised by an exaltation of the vital forces, undergo usually towards morning their chief exacerbations: at the same period too their inversion most frequently takes place.

Catarrhal and mucous fevers, which are generally characterised by the slowness of their movements and the atony which accompanies them, commence most frequently, and also become most exasperated, on the approach of night.

Bilious fevers, which seem to occupy a place between inflammatory and mucous diseases, have their paroxysms as well as their most frequent invasion about noon.

The period of the paroxysms approaches to either the morning or the evening, according as the prevailing diathesis is of a sthenic or of an asthenic character.

It is during the day that by far the greatest number of accessions and paroxysms of intermittent fevers occur. On the other hand, the exacerbations of hectic fever usually occur in the evening and during the night: the sweats take place almost exclusively towards the morning.

*Ramazzini,* in his account of the epidemic constitution of 1690, describes an ataxic remittent fever, of which all the symptoms became greatly aggravated on the approach of sunset. The patients were alarmingly ill during the whole night; but from the first appearance of the sun, all the bad symptoms ceased, and the patients could rise and walk about, *velut angues ad solem, cutem curantes, erecti*—to use the expressions of the writer.

*Huxham* too, in his beautiful treatise on malignant angina, remarks that this disease, during its entire progress, presented exacerbations during the evening; and that, even when the patients were tranquil and comfortable during the day, the symptoms always became aggravated in the evening.

We may here, with advantage, allude to a curious approximation, that has recently occurred to our minds, between the facts now mentioned and the beautiful discovery of M. *Daguerre.*

According to the observations of this gentleman, the hours in the morning and in the evening, equidistant from noon and corresponding, therefore, to the same amount of elevation of the sun above the horizon, are not, however, equally favourable for the production of photographic images. He has found that at all seasons of the year, in circumstances of the atmosphere apparently exactly similar, the image is formed a little more quickly at seven o'clock in the morning, for example, that at five o'clock in the afternoon, at eight a.m. than at four p.m. and at nine a.m. than at three p.m.

Now, if such be the case with the action of light on dead matter, there may be something analogous to it in its influence on living bodies both in disease and in health. The human frame, it is well known, is more sensitive than the most delicate instruments that can be made: why then may it not be acted upon in some peculiar way by the ever-varying conditions of the light and heat of the sun? It is, therefore, quite possible that the discovery of M. *Daguerre* may give rise to the discovery of many phenomena in physiological and medical science.

The great fact of the diurnal movements is seen not only in some acts of human life; but also in many phenomena observed among the lower animals. But the same law does not apply to all, or give rise to uniform results: for the influence of light and heat is often manifested in a manner that is different and even quite opposite, not only in individuals, but even in tribes both of the animal and of the vegetable kingdom. There are some nocturnal species of animals among mammals, birds, reptiles, and insects.

The same phenomenon of diurnal movements is observed in several functions of vegetable life. Every one has heard of the sleep of plants, Flora's clock, &c. &c. *Draparnaud* has observed that at the end of Autumn, when the weather begins to become cool, the flowers of the ipomœa violacea, and of several species of mirabilis, which are usually night-blowing flowers, open at this period of the year during the day also.

Analogous diurnal movements have been observed in various phenomena of meteorology; as, for example, in the indications of the compass and the barometer. It is at the hottest hours of the day that hail is generated most abundantly, and in Europe it almost always falls during the day.

It is probably by following out such analogies as these, in bringing them together, and in comparing them, that we may hope to discover the laws which these phenomena obey, and the general causes which give rise to them.— *Gazette Medicale.*

---

### Fatal Effects from the Use of Colchicum and of Tartrate of Antimony.

*Case* 1.—A middle-aged woman, whose health previously was very good, was seized, in the Spring of 1839, with acute rheumatism. She was bled, and the following prescription ordered:—

℞. Tinct. vinos. colchici ℥i.
Infusi chamomill. ℥iv.
Aq. laur. ceris. ʒi.
Syrupi simpl. ℥i. M.

A spoonful to be taken every two hours.

The medicine produced at first severe vomitings and purgings, which weakened the patient's strength so much, that the third day she refused to continue its use. After nearly a month's omission, its use was resumed; but again it excited great gastric and intestinal disturbance, although a spoonful was taken only thrice a day.

The vomiting became now unusually violent and distressing, and continued so even after the use of the medicine was omitted. This unpleasant symptom became again aggravated by the colchicum having been on one occasion administered in an enema, and at last it was so obstinate that it resisted every means used to check it, such as leeches and anodyne poultices to the stomach, Seltzer water, Riviere potion, aerophorous powder, magnesia, opiates, ice, antimonial plaster, blister, endermic use of morphia, mercurial opiate frictions, &c. &c.*

All proved ineffectual; and the extreme irritability of the stomach seemed to be only aggravated by every thing that was put into it.

"It was no longer a question of rheumatism, but of increasing debility and inanition from the obstinate vomiting and purging which continued. It is true that from the 5th of April—(the first dose of colchicum was administered on the 15th of February preceding)—I had tried every means to support the patient's strength with enemata of beef-tea, and with gelatinous baths. (!) Towards the end of April the vomitings, which returned irregularly once or twice a day, became of a brown colour, and sometimes bloody; and to the state of constipation, which had existed for some time, succeeded a diarrhœa which resisted every means to check it. On the 14th of next month—three months and a half after the commencement and of her illness, and about two months after the administration the second time of the colchicum—the patient died.

*Dissection.*—The stomach only was permitted to be examined. The only morbid appearance found in it was a large patch of inflammation to the extent of four inches at the great extremity of the viscus: the mucous membrane at this part was red, injected, slightly swollen, and somewhat softer than the rest. But altogether the existing lesion was less considerable than what is often met with in the stomach in cases where its functions had not been disturbed."

Dr. *Forget*, the Professor of Clinical Medicine at Montpelier, and in whose practice the preceding case occurred, candidly acknowledges that the *colchicum* was the *point de depart* of the fatal symptoms. He seems to be astonished that the distressing accident should have occurred, seeing that he has long been in the habit of giving the colchicum in such doses as to induce severe vomiting and catharsis,† and that these symptoms have always subsided on the suspension of the medicine.

*Remarks.*—It certainly appears to us that the treatment in the preceding case was most injudicious. What possibly should induce a physician to persevere in the use of any medicine when it produces such distressing effects as the colchicum did from the very commencement of its administration in this case of Professor *Forget?* Not to allude to the immoderate doses in which it was at first administered—nearly a drachm of the vinous tincture every two hours—there are surely other medicines and other modes of treatment suited for the cure of rheumatism, which a prudent practitioner would resort to if he found that col-

---

* No mention is here made of two of the most powerful remedies for allaying the irritability of the stomach—hydrocyanic acid, and creosote. It is unnecessary to suggest these to the British practitioner, as every one is in the habit of using them for such a purpose; our only motive being to allude to the apparent want of practical skill in some of the best foreign physicians. There are two other means which we have often found of decided efficacy in subduing extreme irritability of stomach, which it may be worth while to mention—viz. the entire abstinence from every thing, food as well as medicine; and the use of enemata, so as to induce an action of the stomach and bowels *downwards*.—(*Rev.*)

† He mentions that he has known it cause fifty evacuations a day, when not more than three table-spoonfuls of the prescription—which we have given above—have been taken in four and twenty hours. A pretty good specimen of heroic treatment!

chicum decidedly disagreed with his patient. There is that strong predilection in so many French practitioners to carry every line of practice and every remedy *a l'outrance*; as if the object of our art was rather to be continually making experiments on patients than to cure them of their diseases. We must candidly confess that the more acquainted that we have become with the results of French practice, as recorded in their own journals, the more we are convinced that not a few of the inmates of their hospitals are sent to their long accounts by the heroic practice which they are made to pass through, and who might in all probability have recovered, had some homœopathic doctor had the charge of them—in other words, had nothing at all been done.—(*Rev*)

*Case* 2.—*Death from an overdose of Emetic Tartar.*

A man, 28 years of age, was admitted into the hospital at Strasbourg, with troublesome cough, attended with sharp pain in the chest and occasional hæmoptysis. The diagnosis of the case at the time was "general bronchitis, chronic induration of the left lung, probably with tubercles and even cavernous excavations, disguised by the quantity of mucus present." A blister to be applied to the left arm, a grain of opium at bed-time, and a mixture containing tincture of digitalis were ordered. Subsequently the acetate of lead—in doses of, at first, a grain, and subsequently of six grains daily—was given.

"As the bronchial discharge, however, continued unchecked, and the patient's strength was greatly reduced, we took the resolution to try a remedy which in some analogous cases had produced decidedly good effects—we mean the tartrate of antimony in a full dose. We had in view the bronchorrhœa; and as to the supposed tubercles, (the existence of which formed in our opinion a contra-indication) we allowed ourselves to be influenced by the advice of *Hufeland*, who recommends, in cases of mucous phthisis with obstruction of the air-tubes, the use of emetics. We therefore ordered our patient a six ounce mixture, containing six grains of emetic tartar and two drachms of syrup of poppies: a spoonful to be given every hour."

The medicine, we are told, produced vomiting only twice, and purging three times; but the effects on the poor patient were most disastrous. On visiting him next day, his pulse was scarcely perceptible, his extremities were cold and pale, and his looks vacant. Two hours after the visit, he expired.

Dr. *Forget*, in his comments on this case, repeats the same remark that he made in reference to the former case—viz. that he had frequently administered the antimonial in as large doses without its causing any unpleasant effects. "Besides," says he, "there was neither excessive vomiting nor purging in the present case, nor asphyxia, nor in short any thing which could account for the death by the ordinary mechanism." In his anxiety to give some plausible explanation of the unhappy accident, Dr. *Forget* bethinks himself of *un genre de mort*, hitherto not well understood by medical men, and the cause for which was first clearly explained by M. *Bouillaud* to consist in the formation of coagula within the cavities of the heart during life. Now, in the present case, both ventricles and auricles were, he says, found on dissection to be filled with coagula, which extended some way along the great vessels, and were also intimately enmeshed in the calumnæ carneæ. "The *stasis* or retardation of the blood caused by the nausea and depression from the action of the antimonial, increased, no doubt, the tendency which existed to the deposition of coagula within the heart's cavities."

*Remarks.*—It is almost unnecessary to append a single word in the way of comment, or rather of entire condemnation, on the practice pursued in this case: it was altogether unwarrantable.

A prudent practitioner would hesitate before giving even moderate doses of tartrate of antimony to a patient exhausted by pulmonary disease; and certainly he would never think of administering this rather unmanageable medicine in

*heroic* doses. We have no wish to press M. *Forget* too far, and we shall therefore close these few words, with the following very judicious remarks from his own pen.

"Such unfortunate cases as the present should have the effect of teaching medical men to proceed with caution, and to examine minutely the morbid individualities before having recourse to medicines *heroiques pour guerir, heroiques aussi pour causer la mort.* Moreover they speak loudly in favour of more gentle and simple remedies which, although they act slowly, can never expose the physician to the remorse of having committed an involuntary homicide." (!)

Such are the very expressions of Professor *Forget* himself: we may leave them therefore without further comment.—*Bulletin de Therapeutique.*

---

### MEMORANDA ON INSANITY.

The following summary of the pathology and treatment of insanity is from the pen of an experienced physician, M. *Bottex,* an inspector of lunatic asylums in the South of France. It is clear and succint, and conveys a good idea of the opinions of one who has seen much of the disease which he professes to describe.

1. Mental alienation, like all other diseases, is the result of a lesion of the organism. The affected organ is the brain—sympathetically at first, but essentially so if the disease continues.

2. The nature of the lesion varies according to the character or form of the existing insanity :—for under this *generic* term, diseases, which have little or no relation to each other, except in the disturbance of the intellectual faculties and which are fundamentally different, have been classed and grouped together.

Thus *idiocy* and *imbecility,* which result from an imperfection of the brain, congenital or acquired, are not, properly speaking, diseases, and require no medical treatment.

*Mania,* which is characterised by a general delirium with excess of action, is produced by an irritation of the outer surface of the brain and of its membranes, in a more or less considerable extent: if this irritation or inflammation does not terminate in resolution, it gives rise to certain organic changes, which induce *dementia* or mental alienation—a state of the disease which is little susceptible of cure. *Dementia* is therefore *mania* become incurable.

*Mania* and *dementia* are often complicated with a paralysis of a greater or less number of the muscles of animal life: this form of paralysis is generally fatal, and seems to arise from a softening of the cortical matter of the brain.

4. *Monomania,* accompanied with excess of action, is produced by a very circumscribed irritation of a portion of the brain and of its membranes. When it is accompanied with depression and melancholy, there is probably neither irritation nor inflammation of any portion of the brain, but only a habit on the part of the invalid of associating incoherent ideas. (This is certainly not very satisfactory nor indeed is it quite intelligible). Perhaps indeed there exists in such cases, much more frequently than is imagined, a chronic lesion of the trisplanchnic nerve, whose disturbed functions may re-act on those of the brain.

5. It is evident that the rational treatment of the various forms of insanity must be based on a knowledge of the pathological conditions of the nervous system with which they are associated. To be satisfied with merely attacking secondary and sympathetic symptoms would be,—in the words of M. *Georget*—like trying to put out a great fire, by extinguishing the cinders and hot ashes which are thrown to a distance by the wind, instead of exerting all your efforts to bear upon the centre of the conflagration.

To *Pinel* we owe a great deal, not only for his having abolished the barbarous practice of treating the insane rather as criminals who deserve to be punished, than as patients who are labouring under a disease, but also for having exploded the use of a vast number of empirical remedies which, before his time, used to be resorted to. He perhaps carried the *expectant* system of treatment too far, by recommending an almost unlimited reliance upon the curative efforts of nature; but certainly this was better than the opposite practice of leaving nothing at all for her to do. It is always a most valuable precept in our art, *primo non nocere.*

6. The treatment of insanity is either physical or moral. The most important *physical* remedies are bleeding, baths, purgatives, and the use of artificial discharges, and they are chiefly useful in *mania*; whereas the *moral* means—under which head we comprehend cheerful occupation of the mind in gardening, music, and games of chance, the engaging in religious services and ceremonies, and in whatever has a tendency to tranquillise and exhilarate the mind, and withdraw it from its delusions—are most to be trusted to in the various forms of monomania.

In reference to the former, or the physical remedies, M. *Bottex* especially recommends the employment of *baths*—not baths "of surprise," but tepid baths, in which the patients should remain for a length of time. These should be used frequently, and they may be most beneficially combined with the application of cold to the head at the same time.

The purgative which is suited above all others for insane patients is croton oil,—of which a drop or two, blended with honey or syrup and then mixed with any fluid, may be easily given to any patient.

When the disease resists the use of bleeding, baths, and purgatives, M. B. recommends that an issue be at once established,—in the thigh in women whose menstruation is irregular, and in the nape of the neck in all other patients. He prefers an issue made with the cautery or with caustic to a seton, as he is of opinion that the very effort made by the part to throw off the eschar exerts a powerfully derivative influence.

In some cases the use of the antimonial ointment rubbed upon and around the scalp, so as to bring out a crop of pustules, has seemed to effect a cure in cases that had resisted the employment of all other means.

The use of sedative narcotics, as morphia, digitalis, and hydrocyanic acid, is frequently productive of excellent effects. Quinine also, especially in cases where the paroxysms seem to have somewhat of an intermittent character, has been found extremely useful.

Each case, be it remembered, requires some speciality in its treatment; and the adaptation of the remedial means to the varying conditions of different patients is the best test of the physician's discrimination and skill.

All the best writers on insanity insist upon the necessity of the separation of the sufferers from their relatives, and from whatever has the effect of keeping up a remembrance of their former feelings and habits. A change of scene is always beneficial; and hence, whenever there is any promise of convalescence, the physician will do well to recommend travelling.*

---

* Dr. Esquirol observes: "I have often found that insane patients are greatly tranquillized by travelling, especially when they visit countries whose striking scenery takes hold of their imaginations, and when they are exposed to the little troubles and casualties of common travellers. The very annoyances and inconveniences to which they are exposed have a good effect. Travelling acts beneficially also by exciting the assimilative functions; the appetite, the sleep, and the various secretions become more regular and natural. Besides the chagrin, which is almost always experienced by the sufferer when on his recovery he returns to his home and friends, is much mitigated after a long travel or voyage, the incidents of which furnish so agreeable a topic for conversation."

So important too is exercise in the open air considered by many, that Dr. *Ferres* has very justly remarked that a farm or large garden is an indispensable adjunct to every lunatic asylum.—*Gazette Medicale.*

---

M. BAUDENS ON GLANDULAR SWELLINGS.

M. *Baudens*, one of the most distinguished military surgeons in France, and who has been the surgeon of the hospital at Lille since he returned from the expedition to Constantine in Africa, where he was the *chirurgien en chef*, prefaces his remarks on the development of glandular engorgements by alluding to the influence of climate on this and other forms of disease.

"Under the cold and humid atmosphere of Lille and of the surrounding country, the energy of the exhalent vessels seems to be quite paralysed, while at the same time the absorbents of the surface, being kept as it were in a perpetual bath, are constantly receiving extraneous matters. Hence the retropulsion of the fluids from the surface by a centripetal movement, the abnormal development of the cellular tissue, the tendency to enlargement of the lymphatic glands, the disposition to rickets, phthisis, and scrofulous disease in all its forms, which so widely prevail among the population of Lille."

It seems that the moisture of the atmosphere at Lille is, as appears from the hygrometer, at least as great again as that at Paris.

M. *Baudens* alludes to the damp state of the houses, the neglect of flannel clothing, and the insufficiently nutritious diet of the people, as powerful adjuvants in relaxing the general constitution, and predisposing it to the class of maladies above-mentioned. "The Englishman," he adds, "who lives in a cold and foggy climate, braces himself against its hurtful influence with his *rosbif;* we should imitate his example."

He attributes much of the disease among the troops to the insufficient allowance of nutritious animal food to the men. Want of due bodily exercise is another reason:—

"The movements of the limbs in active exercises, and the continued occupation of the mind, must very efficiently second the good effects of a more abundant and more animalised nourishment. A proof of this we see in the case of cavalry soldiers, who, in consequence of their more active and energetic employments, are much less frequently on the sick-list with glandular complaints than the infantry soldiers."

The influence of the seasons on the development of this class of diseases is also great.

"Tolerably rare during the three Summer months, they re-appear with the fogs, cold, and dampness of Autumn; but it is chiefly during Winter that they increase with such a prodigality that we have often counted nearly 80 patients in our military hospital, which usually contains only 300 sick, affected with inflammatory glandular enlargements (*adenites.*)"

The groupes of glands most frequently inflamed are the cervical and maxillary: occasionally the testicle has been similarly affected at the same time; and in not a few cases this gland has begun to swell and become painful as the lymphatic glands began to subside; while in others the orchitis was primary and the affection of the cervical glands was secondary. The management of these varieties of the disease is sufficiently easy during the early stage: the use of antiphlogistics, purgatives, and sudorifics, generally suffices to remove all such inflammatory swellings. The difficulty consists in effecting a resolution of the glandular tumors, when they have become fairly chronic.

M. *Baudens* prefers the use first of blisters over the part to any other local remedy, and then of ioduretted mercurial embrocations. Compression is often a most valuable adjuvant. The following *novel method* of applying this in swellings of the testicle has succeeded in the practice of M. *Baudens.*

"I had the gland inclosed in a bottle of caoutchouc, which by its elasticity kept up so steady and methodic a compression that at the end of a month's use I had the gratification of seeing a radical cure of the enlarged testicle—which had been diseased for two years, and which would certainly have been extirpated if it had not been that the inguinal glands were seriously affected at the same time."

M. *Baudens* mentions favourably the hydrochlorate of barytes and the preparations of iodine, as useful remedies for internal administration. A generous diet should be allowed at the same time. Whatever tends to fortify the general system, and to give tone and energy to the blood-vessels, will be found to promote the success of our remedial treatment. It is often of marked service in obstinate cases to recur to the application of blisters, often omitting their employment for some time.—*La Lançette Française.*

---

### On the Treatment of certain Cases of Deafness.

M. *Petrequin,* surgeon of the Hôtel Dieu at Lyons, has communicated to the French Medical Gazette a lengthened paper on those cases of deafness which are connected with, and not unfrequently induced by, affections of the throat and nostrils. He first discusses the use of the different parts of the ear;—take, for example, what he says of the uses of the Eustachian tube.

"It serves as an excretory canal for the fluid secreted within the tympanum, which would otherwise accumulate and interrupt the sense of hearing, (as is the case when the tube becomes obstructed from disease); hence this office justifies its appellation of aqueduct of Eustachius.

2. It serves to establish a uniform degree of heat in the air contained in the tympanum, and thus prevents the disturbance of the functions of the component parts of this cavity.

3. It serves to maintain in this air its proper hygrometric condition.

4. Its principal use is to keep the air in the tympanum in a state of equilibrium with the external air, so that its membrane may vibrate accurately, and communicate the impulses of the air on each vibratory movement.

It is well known that a common drum, in order that it may give out a proper sound when played on, must have an opening in its side to give entrance and exit to the air; so it is with the drum of the ear; if the Eustachian tube be obstructed, the membrana tympani ceases to vibrate with the impulses of sound, and thus the sense of hearing becomes more or less entirely obscured.

The circumstance too of many deaf people hearing better when their mouth is open may be accounted for by the readier admission which is thus given to the air, along the Eustachian tube, and also by the slight enlargement of the outer auditory passage, in consequence of the motion of the condyles of the jaw.

Again; it is from the admission of too large a quantity of air along the tube in the acts of yawning and sneezing that sometimes occasions a momentary deafness and sense of tickling in the ear; whereas, after very loud sounds, such as from the explosion of fire-arms, we not unfrequently experience a tickling in the throat and mouth—in consequence of the air within the tympanum being driven outwards with unusual force. Hence cannoneers are often in the habit of keeping their mouths open while firing off artillery.

From all these circumstances it may be readily understood how any morbid affections of the tympanum and of the Eustachian tube must necessarily interfere with the sense of hearing. Now the Eustachian tube very generally suffers in all chronic diseases of the pharynx and throat; and hence the importance of examining the fauces in all cases of deafness."

M. *Petrequin* details the particulars of numerous cases where the deafness had been preceded or was attended by some chronic inflammation of the throat, and in which a cure was effected by the use of alum gargles, of insufflations of a powder consisting of equal parts of alum and sugar twice a day, and occasionally by rubbing the fauces with a stick of alum.

The deafness, arising from an obstructed state of the Eustachian tube, may generally be suspected whenever it has followed upon any affection of the throat. It is most commonly met with in persons who are subject to catarrh, cynanche, and coryza; also in those who have suffered from syphilitic, dartrous, or rheumatic complaints. The affection of the tube is necessarily much more obstinate than that of the throat, and it often exists quite unsuspected. The deafness caused by it usually varies in degree according to the state of the weather and the season of the year, being always greater when the air is cold and moist, and less decided when it is dry and warm. A fit of sneezing or of yawning too will often relieve the deafness more or less, for a short time, the patient experiencing at the moment a sensation as if a bottle was uncorked in his throat: this may be caused by the expulsion of some firm mucus plugging up the tube. This kind of deafness is always much increased, if coryza or any form of cynanche is present; and lastly, we may add that, "if during a strong expiration, while the mouth and nostrils are closed, the person is not sensible of the air passing along the tube and distending the tympanum, we have good grounds for suspecting that the former is obstructed."

Deafness from this cause is not unfrequent in youth and in middle age; perhaps however it is still more common in old people, as it is well known that in them few of the mucous passages are quite exempt from some morbid change.

In cases which resist the treatment recommended above, the catheterism of the Eustachian tube should be practised at the same time: this operation is far from being so difficult as many writers have imagined. But very generally M. *Petrequin* has succeeded with the use of the alum, in the form of gargle, and of dry insufflations to the back of the throat. The gargle should be used frequently in the course of the day, and be retained in the mouth for a considerable length of time, so that the alum may affect the mucous membrane, and its operation be gradually communicated towards the opening of the posterior fauces. The insufflation of the dry powder, consisting of equal parts of pulverised alum and sugar, should be employed twice a day; and to render its operation still more effectual, it will be found very useful to rub gently with a stick of alum the tonsils, velum, and fauces, every three or four days.

Under the influence of these several means the engorgement of the throat gradually subsides, and a more healthy action of the mucous membrane is established.

"Experience has already testified in favour of the aluminous medication; MM. *Pommier* and *Bretonneau* have recommended it strongly in cases of diptherite, M. *Bennati* in various affections of the vocal organs, M. *Velpeau* in diseases of the throat, and I myself have used it with great success in many forms of ophthalmia."—*Gazette Medicale.*

*Remarks.*—M. *Petrequin* is quite correct in ascribing numerous cases of deafness to affections of the Eustachian tube, and in stating that these affections are very often primarily attributable to a chronic inflammation of the fauces, which has gradually extended itself upwards to the posterior nostrils. An excellent, and a still more potent, substitute for the alum, as recommended by M. *Petrequin*, is the nitrate of silver. This is to be used in the form of solution, and the posterior fauces should be freely wetted with it, every three or four days, by means of a sponge fastened to a probang. A gargle, containing the hydriodate of potash, has also in several cases been used with decidedly good effects.

The internal administration of the same salt, along with sarsaparilla, may be most advantageously combined with the employment of local means, when the constitution is decidedly scrofulous, or suffering from the effects of the syphilitic disease.—*Rev.*

---

### Remarks of a German on English Obstetricy.

"In general the English physicians are much less disposed than their brethren on the Continent to perform operations, and trust more on all occasions to the efforts of nature. This will appear when I mention that *Clarke,* during the seven years that he was accoucheur at the Dublin Institution, states that in 63 cases only out of 10,199 deliveries was the employment of any instruments resorted to, and that *Collins,* the late master of the same extensive charity, had recourse to them only 145 times in 16,654 cases. This unwillingness to employ any forcible means, unless peremptorily required, arises no doubt from the slight importance attached by the English to the life of the infant compared with that of the mother. Hence the frequency with which embryotomy is performed, and the comparative rarity of the use of the forceps; and, as to the Cæsarian operation, that is never thought of, except where the child cannot be extracted even after the head has been perforated. (Strange that it ever should.—*Rev.*)

As to the mortality among the infants, 1121 out of 16,654 were born dead during the mastership of Dr. *Collins.* Of 97 cases in which there was prolapsus of the umbilical cord, 73 of the infants were born dead; the mothers did well in all these cases. Dr. *Clarke,* the predecessor of Dr. *Collins,* has told us that only 17 children out of 66 cases, where there was prolapsus of the cord during labour, were born alive. The mortality among the women seems to be very small; for of 16,414 who were delivered in the institution during Dr. *Collins'* mastership, only 164 died; although during this time there had been an epidemic of puerperal fever.

In cases of uterine hæmorrhage, the English accoucheur relies chiefly on a speedy extraction of the placenta after the child has been delivered: in usual, the placenta is never allowed to remain longer than one hour after the accouchement. The employment of firm compression of the hypogastrium, of cold applications or douches, and the introduction of the hand into the cavity of the uterus, are generally resorted to in this form of hæmorrhage. What are considered by far the most potent internal remedies are opium and spirituous drinks, whenever there is any tendency to syncope.

It is only in the last extremity that Mr. Kennedy has recourse to the use of the forceps in the case of puerperal convulsions: he very generally trusts to the efforts of nature. In 15 out of 30 cases of convulsions—and of these 24 were first-labour cases—the delivery was spontaneous; in six it was effected with the aid of the forceps; in eight the perforator was used; and in one only turning was performed. Of the 30 children, 14 only survived. Of the eight women, in whose case embryotomy was performed, five died."—*Neue Zeitschrift fur Geburtskunde.*

---

### On Epidemic Hemeralopia.

Dr. *Henry,* surgeon of the French Frigate Dido, which has recently returned from its surveying voyage round the world, has communicated some observations on this species of amaurotic blindness, which is not unfrequently met with in tropical climates. The following extracts are from a report made by him to the Council of Health at Brest.

"A remarkable circumstance occurred during our voyage from St. Domingo to Martinique; it was the epidemic appearance of a complaint of frequent occurrence in the tropics, *hemeralopia.* A few scattered cases had indeed already appeared during the previous part of the voyage; but now a number of our crew became almost suddenly affected with night blindness—in consequence probably of our prolonged stay at sea under the dazzling rays of an equatorial sun."

Dr. *Henry* applied himself to ascertain the real cause of the complaint. He quite rejects the idea of some, that it is the result of any miasmatic influence, for the Dido was and had been for some time at sea; of others, that it is occasioned by imperfect nourishment, as the crew were upon fresh provisions at the time; or by the moisture of the atmosphere, any unwholesomeness of the night air, or by the noxious effect of sleeping in the moonshine (a popular idea). He feels assured himself that the real cause of the blindness is altogether owing to the dazzling effects of the intense solar light reflected from smooth shining objects, such as the sea, tracks of sand, &c. Hence it is more common at Lavalette in Malta and at Cadiz than at Martinique or Guadaloupe, because the two former cities are built upon and surrounded with a sandy coast, whereas the latter have extensive and shady forests all around them.* We cannot therefore hesitate to admit that it is to the intensity of the brilliantly reflected light from the smooth surface of the tropical sea giving rise to an atony of the retina that we are to attribute the sudden appearance of so many cases among the sailors of the Dido at the period of her voyage above mentioned. The following remarks on the symptoms of the disease are interesting.

"The symptoms of night blindness are often very indistinct. Generally indeed the pupils are considerably dilated, and lose in a greater or less degree their contractility; sometimes the eye is painful, at other times it is quite free from all uneasiness. . . . . . We have not observed that this malady is attended, as has been asserted by *Bampfield, Dupunt, Scarpa* and others, with vertigo, headache, redness of the face, or any gastric or intestinal disturbance. In our practice it has presented itself free from all complication, and has almost always been primitive and isolated. The accession of the complaint is usually gradual. At first the sight has become indistinct and obscured by, as it were, a thin veil before the eyes, after the setting of the sun; this indistinctness increases more and more by degrees, and at length terminates in total blindness. I observed that in those patients, in whom the iris was blue or grey, the pupil was always more dilated than in those whose eyes were dark; and moreover that the disease was much more common in the young sailors, who had never been in a tropical climate before, than among the old and seasoned part of the crew."

We need scarcely mention that the complaint is comparatively very rare among the officers of a ship's crew; their duties not exposing them, like the sailors, to the unprotected action of the sun's rays.

As to the *prognosis* in hemeralopia, it is almost always favourable, provided the patient be withdrawn from its exciting cause, and especially if a change be made to some other latitude where the light is less dazzling and overpowering.

"We have observed in between 30 and 40 cases the duration of the complaint has been from eight to fifteen days, which in others the restoration of

---

* Dr. Henry mentions that almost every one of the unfortunate French prisoners of war, so inhumanly left by the Spaniards on the island of Cabrera (Baleares)—where they were badly fed, still worse sheltered, and exposed to the heat of an African sun, on a sandy coast where there was little or no vegetation—became affected with hemeralopia.

healthy vision was much more rapid. As this restoration took place, the contractility of the pupil gradually returned, and the expression of the features lost that dull and unintelligent aspect which it had acquired."

As to the *treatment* of hemeralopia, the protection of the eyes from the brilliant sunshine is an indispensable preliminary, although this alone will not suffice for the removal of the blindness. The application of a blister to the temple or to the nape of the neck is one of the best remedies that can can be employed. The use of gentle purgatives should not be neglected at the same time.

Dr. *Henry* states that, even under favourable circumstances, the employment of ammoniacal vapour, and of other local stimulants alone, did not produce much good.

The fumes arising from boiled liver, so strongly recommended by *Dupont* and others, did not appear to have any peculiar advantage.

Dr. *Henry* recommends that the men should be kept as much as possible under the awnings, and when they are exposed to the sun's rays, that they should be made to wear a straw hat with a wider brim than sailors generally use, and which should be covered with some green-coloured lining.—*Gazette Medicale.*

---

### M. Guerin on Subcutaneous Wounds.

Every one has heard of the proposal, or rather now the practice, of M. *Guerin* of Paris, in the treatment of lateral curvatures of the spine—the subcutaneous division of several of the muscles of the back.

From what he recently stated to the Royal Academy, he has met with extraordinary success. " I have, in nearly fifty cases, divided more or less completely most of the muscles of the back and spine—viz. the trapezius, the rhomboideus, angularis scapulæ, sacro-lumbaris, and longissimus dorsi. In each of these operations, the wound of the muscle has been from three to four inches in length, and sometimes from one and a half to two and a half inches in depth, when the sacro-lumbaris and longissimus dorsi muscles were completely divided: in those cases where the trapezius and rhomboideus were entirely cut across, the bistoury traversed at least from four to five inches, or more, under the skin.

In almost every one of these cases, no local inflammation nor constitutional feverishness supervened, and by the third day many of the patients were able to rise up and walk about without support. This most pleasing exemption from the accidents which so often follow upon ordinary wounds, is entirely attributable to the circumstance of the air not coming in contact with the freshly-divided surfaces: the tendency to inflammation seems scarcely to exist, and the parts commence and complete the reparative process without interruption or pain."

In addition to a multitude of experiments performed on dogs, M. *Guerin* has established the perfect innocuousness of subcutaneous wounds in operations for the relief of various forms of distortion. He has divided the sterno and the cleido-mastoid muscles (separately, simultaneously or consecutively) for wry-neck twenty-five times: in every one of these cases—the section having been made under the skin—the wound healed without the smallest trouble. Occasionally the operation was effected with considerable subcutaneous effusion of blood; but this was usually absorbed in twenty-four hours or so, and the reparative process then rapidly took place. Again, the operation of dividing the tendo-Achillis for club-foot he has performed upwards of 200 times; and in all these also he has been equally successful. In the practice of other surgeons, indeed, inflammation and suppuration has followed this simple operation; but these troublesome consequences are, we suspect, always owing to the outer wound having been made too large, and the air having thereby been permitted to enter.

"That such is the case is surely sufficiently proved by the success which has attended my operations. I have now performed the subcutaneous section of different tendons and muscles in upwards of 500 cases; and in not one instance have any troublesome inflammation of the wound supervened."

The great object, therefore, of the surgeon should be to prevent the entrance of the external air into the wound.

"From all these circumstances," continues M. Guerin, "I infer that subcutaneous wounds so quickly heal in consequence of the exclusion of the external air; and that it is from this fluid (the air) neither physically obstructing the circulation, nor chemically modifying the properties of the blood, nor altering in any degree its vital constitution, as well as from its not exerting any hurtful influence on the nerves and other parts which are protected from it, that the lacerated or divided tissues coalesce and unite by the first intention without any of the usual inflammatory symptoms."

That there is much truth in this observation is apparent from the surprising rapidity with which extensive effusions of blood are absorbed, and laceration of the soft tissues after some dislocations and bruises are healed, when there is no outward wound communicating with the seat of the injury. The great source of danger in compound fractures and dislocations is doubtless in the exposure of the lacerated parts to the action of the atmosphere. The dangerous effects of the admission of the air into the sacs of large abscesses is also well known to every surgeon.

*Postscript.*—The principle of subcutaneous division may be advantageously applied to other operations, besides that of the section of contracted muscles and tendons in cases of deformity.

In a recent number of the *Gazette Medicale*, we observe that M. *Barthelemy*, surgeon of the Hospital *Gros Caillou* at Paris, has strongly recommended this method of dividing synovial tumors. He slides a longish narrow-bladed scalpel under the integuments, and cuts the tumor fairly across in the middle, so that all its contents must be extravasated into the surrounding cellular tissue: the knife is then withdrawn by the small puncture, and firm compression is made on the part for a few days.

M. *Barthelemy* mentions three cases in which this mode of treatment has been quite successfully adopted. He suggests also, that perhaps other kinds of tumor may be advantageously treated in the same manner.

M. *Malgaigne* also has adopted in one case, where there were several ganglions or synovial swellings over different joints, the method of dividing the tumors fairly across from side to side under the integuments. He suggests its applicability to the treatment of some cases of hydrocele.—*Gazette Medicale.*

---

### M. Guerin on Club-foot, Wryneck, &c. &c.

In a lecture which this gentleman recently gave of the results of his clinical practice, during a period of three months, in the treatment of various sorts of deformity, he thus explains his views as to the primary cause of club-foot, and of congenital irregularities of the joints, as well as of some kinds of deformity affecting the spine.

This cause, in his opinion, is the contraction, acting unequally and irregularly, of the muscles of the part.

"All congenital articular deformities are, like club-foot, the effects of convulsive muscular contraction; and the various sorts of these deformities are the results of the combinations of this contraction differently distributed in the muscles of the trunk and of the limbs."

In confirmation of the truth of this opinion, he alludes to the state of the muscles of the calf in cases of club-foot: they are hard, unyielding, and, as it were, matted together; the tendons are stiff and projecting, and so tense as to resist all attempts to restore the joint to its right position. The muscles chiefly affected in club-foot vary in the different varieties of this deformity. Thus when the foot is twisted outwardly, the tibiales, anticus and posticus, are the most contracted; when it is turned inwardly, the tibialis posticus, the adductor, and the flexor pollicis are at fault; in cases of valgus with abduction of the foot, the interior and lateral peronei are chiefly affected; and in complex club-foot, the extensors and the flexors of the toes are all more or less unnaturally contracted. In addition to the irregular contraction of certain muscles, there is in some cases, at the same time, a greater or less degree of partial paralysis.

The following table will indicate, in a certain degree, the comparative frequency with which different muscles of the leg are apt to be affected in club-foot:—

| | | | | | | |
|---|---|---|---|---|---|---|
| In 17 | cases, the tendo Achillis was divided. | | | | | |
| 12 | ,, | the tendon of the tibialis ant. | | | | was divided. |
| 3 | ,, | .. | .. | .. | ,, post. | ,, |
| 1 | ,, | .. | .. | .. | extensor dig. | ,, |
| 4 | ,, | .. | .. | .. | extens. pollic. | ,, |
| 1 | ,, | .. | .. | .. | peroneus ant. | ,, |
| 3 | ,, | .. | .. | .. | flexor digitor. | ,, |
| 3 | ,, | .. | .. | .. | flexor poll. | ,, |
| 2 | ,, | .. | .. | .. | abductor poll. | ,, |
| 3 | ,, | .. | .. | .. | flexor brevis dig. | ,, |
| 5 | ,, | .. | .. | | the aponeurosis plantaris | ,, |
| 54 | | | | | | |

"You thus see, gentlemen, that the tendons of every muscle of the leg, with the exception of the lateral peronei,* have been divided. Conformably to our principles, we have attacked each variety of the deformity, by the section of the tendon or tendons producing it. In no one instance have we failed: you have seen successively disappear the different elements of each deformity by the practice that has been pursued. Where my predecessors were satisfied with restoring the normal *direction* of the foot by the division of the tendo Achillis, we have laboured to effect the complete restoration of its *forms*, the removal of the *curvatures* of the plantar arch, of the forced abduction of the foot, of its curvatures inwards, of the subluxation of the toes, and of the twisted position of the foot—elements, the cause and mechanism of whose production were quite misunderstood, and the proper treatment of which had been quite overlooked."

M. *Guerin* next alludes to the various auxiliary means, such as manipulations, bandages, plaster of Paris moulds, and machines, used in the treatment of club-foot and other such like deformities.

He admits that each of them is respectively useful in certain cases. By well applied manipulations, after the contracted tendon or tendons have been divided, the foot may sometimes be at once restored to its right position almost as speedily as when a dislocation is reduced. When, however, the resistance is

* These muscles were divided in a case which occurred subsequently to the delivery of the lecture.

much greater, then recourse must be had to bandages, instruments, and plaster moulds. M. *Guerin* speaks in terms of high praise of the last-named means.

"When the deformity has been very great, and the resistance is so decided as to prevent the adjustment of the limb either with the hand alone, or with the aid of instruments, (after the section of the contracted tendons) or when the skin is so tender that a sufficient degree of mechanical compression cannot be employed without risk of injury, then the plaster mould, by distributing an equal amount of pressure on every point of the limb, and by retaining and concentrating on its surface the cutaneous exhalation, is by far the best means for overcoming the resistance, and repairing any injury to the integuments which may have been caused by the use of either bandages or instruments. Such circumstances have several times occurred in our practice during the treatment of the extreme and complex sorts of club-foot, in children of from two to four years of age."

The period of time required for the cure of club-foot varies a good deal according to the duration, and the particular form or variety of the deformity that exists. About four weeks may be considered to be about the average time required; but some cases do not require above three weeks, and others need nearly three months before a complete cure is obtained. Whenever the case is of a complicated nature, as when the primary or essential deformity is associated with other deviations from the normal shape and direction of the foot, the treatment required will necessarily be not only more protracted and troublesome, but is seldom so perfect and complete as in simple cases.

Besides the cases of club-foot treated at the Clinique of M. *Guerin*, there were ten cases of *wry-neck* admitted. His remarks on this deformity are to the following purport:—

"You now know that the two phases—the ancient and the recent—of this deformity are two morbid states, very different from each other, of the muscular system, and which require different methods of treatment. We have already explained to you that permanent *contraction* (contracture) is very different from *retraction*, and we have insisted the more on this distinction, seeing that no surgeon had attended to it before ourselves. The *former* is the spasmodic shortening of a muscle, without any appreciable alteration of its tissue, so that if the contracted muscle were stretched out, all its normal characters would be at once restored. The *latter*, on the contrary, is that shortening produced at first indeed by contraction, but in which the texture of the affected muscle has become subsequently more or less deeply altered, and has thus assumed a greater or less degree of a fibrous or of a fatty consistence.

Attention to the distinction between these two very different conditions of a contracted muscle is necessary to the establishing of a scientific practice. If there be simple *contraction*, we may hope to effect an elongation of the contracted muscle by means of extension, rubbing, shampooing, &c.; whereas, if there be genuine *retraction*, the only rational method of relieving the deformity consists in dividing the affected muscle.

We have exemplified this distinction, before your eyes, in the treatment of wry-neck, according as the deformity has been of old or of recent formation; and the success which has attended our practice has abundantly proved the correctness of the diagnosis now pointed out.

Among the most useful means to promote the elongation of muscular contraction is the use of friction with antimonial ointment: the eruption which this excites has seemed to me to prepare the way for the more successful employment of extension. In three cases of wry-neck treated before you, the re-adjustment of the head has appeared to be intimately connected with the eruption of the pustules over the contracted muscle."

M. *Guerin* subsequently alludes to the operations of dividing the biceps, semitendinosus, semimembranosus, rectus internus, Sartorius, &c. for relieving the deformities of the knee and hip. He concludes by stating that—

"During the last four months I have performed before your eyes 68 operations of dividing tendons and muscles, in not one of which has any troublesome symptom supervened, and all of which (with upwards of 400 other cases of analogous operations performed by me during the last four years) tend to confirm the truth of my theory of *subcutaneous wounds*, and establish the superior advantages which I have attributed to this method of operating over the usual one."—*Gazette Medicale*.

---

### M. Malgaigne on Herniæ.

M. *Malgaigne* has, during the last season, delivered a series of lectures on herniæ at the central bureau of hospitals in Paris. From these we learn that, according to his statistical enquiries, nearly one-twentieth of the entire population in France are affected with ruptures. It is therefore one of the most important duties of every surgeon to make himself thoroughly master of every practical point on the subject of an infirmity that is of such frequent occurrence. M. *Malgaigne* very justly remarks that professional men have far too much neglected the management of *reducible* herniæ, and have left the construction of trusses altogether in the hands of mere machinists. He says, "a few years ago there was scarcely a surgeon, with the exception of Sir *A. Cooper* and some of his pupils, that knew how to put on a truss properly. The mere bandage-maker was certainly better qualified to advise a ruptured patient as to his truss than the medical attendant. Thus when *Salmon* proposed his apparatus, known in France by the name of the *English bandage*, our surgeons did not at all understand its simple mechanism, and they suggested some modifications which quite destroyed its utility."

M. *Malgaigne* confesses that he knew as little of the matter as any of his confreres, four years ago, when he was appointed examiner of all the herniary patients who presented themselves at the Central Bureau. From that period to the present he has examined upwards of 2000 cases of rupture and of prolapsus; and his attention has necessarily been directed in an especial manner to discover which are the most efficient and serviceable trusses. With this view he selected several cases in which the rupture was the least easily retained, and he shewed them to the various instrument-makers in Paris for the purpose of trying the comparative value of the different forms of trusses in the same patients and under the same circumstances—the only true way of determining their relative superiority. Some of these gentlemen acceded to the proposal with willingness, but others with an ill-dissembled reluctance.

According to the researches of M. *Malgaigne*, inguinal hernia is of much more frequent occurrence even in women—contrary to what is usually stated in books—than crural hernia.

The following observations on the prolapsus vaginæ are novel:—

"It is usually stated by writers that vaginal cystocele is of exceedingly rare occurrence; whereas I find that it is the most common form of vaginal prolapsus. Again, rectocele was not even mentioned in surgical writings a few years ago; and yet it is quite as frequent as the genuine prolapsus of the uterus."

M. M. proceeds next to point out the *relative frequency of herniæ in the male and female sex.*

Of 2767 cases, which he examined during the year 1836, 2203 occurred in males, and 564 in females; or about 4 to 1; and of 2373 cases examined in

1837, 1884 occurred in the former, and 489 in the latter. The relative frequency therefore of hernia in the two sexes may be stated to be as 4 is to 1.

*Relative frequency at different Periods of Life.*

If we endeavour to discover the relative frequency of ruptures during the first year of life to the entire number of cases taken at all ages from infancy to old age, we shall probably find that it may be stated at about $\frac{1}{52}$ of the whole. It is not the same indeed in the two sexes; for it would seem to be about $\frac{1}{38}$ in male infants, and only $\frac{1}{62}$ in female ones. But even this proportion is much higher in the female sex than at all the other periods of life put together. The reason of this is easily given. During the first year of infancy ruptures are almost always either umbilical or inguinal. Now as to the former kind, the one sex is quite as much exposed to it as the other; and, even in respect to the latter, it is necessary to bear in mind that the canal of *Meckel* in female children is as frequently open as the inguinal canal in male ones. It is quite true however that the descent of the testicles must favour the predisposition to rupture at this point, and is to be regarded as the cause of the greater frequency in boys than in girls.

The frequency of ruptures diminishes very sensibly from the first to the second year; and this diminution becomes greater and greater till about the twelfth or thirteenth year. From about this period the tendency begins very sensibly to augment, at least in the male sex; owing no doubt partly to the incipient increase of development in the generative organs, and partly also to the violent muscular efforts which boys employ in their various sports. But it is chiefly from the 20th to the 28th year that the great increase of herniæ in both sexes is most remarkable. In women, exomphalos is now not unfrequent; and crural rupture—before this period of life excessively rare—is also met with. The changes that have taken place in the female pelvis and in its soft envelopes, more especially if the woman has married and become a mother, will at once account for the great increase of ruptures during the above-mentioned period of life. This increase amounts to at least a double; whereas, in the male sex, it does not exceed by one-fourth of what it had been.

From the 28th to the 35th year, the tendency to ruptures in both sexes seems to be stationary; but from the latter to the 40th year it again increases, and this very sensibly too.

From the 40th to the 50th year, it again decreases in men, but rather increases in women.

M. *Malgaigne*, from an examination of the tables drawn up by the medical officers of the army appointed to inspect the young men, between 20 and 21 years of age, who have to serve as recruits every year, estimates that at this period one in every 32 is affected with some form of hernia; that at 28 years of age there is one in every 21; at from 30 to 35 years, that there is one in every 17; and at from 35 to 40 years there is one in every nine. The ratio remains then stationary till about the 50th year; after that it rises to about a sixth; from the 60th to the 70th year to a fourth; and from the 70th to the 75th year to nearly a third of the male population.

With respect to the ratio of the number of persons affected with herniæ to the entire population in France, M. *Malgaigne* states that he estimates it at a one-twentieth.

He next proceeds to examine the *causes*, predisposing and exciting, of ruptures. Among the former, hereditary tendency is one of the most influential. Of 316 cases of ruptures of different kinds he found that in 87 the parents of the persons had been similarly affected. Stature is another: a very large proportion of herniary patients being above the average height. This we might indeed, à priori, expect; as tall men are seldom so robust and strongly-knit together as shorter men.

Alluding to the French army, M. *Malgaigne* observes :—" The regiments of Carabineers, which are the finest men of our army, are perhaps those which can worst support protracted fatigue, and which send most invalids to the hospital. On the other hand, our Hercules of the North are short-built men, who are capable of resisting for a much greater length of time every deteriorating influence."

Nearly twice as many inguinal herniæ occur on the right side as do on the left one. This may be owing to the greater effort of all the muscles on the right side, and also to the larger size of the right testicle and spermatic cord than of those on the left side.

The greater frequency of herniæ on the right side applies to infants as well as to adults. In the case of the former, the reason will probably be found in the circumstance that the right inguinal canal is (according to the observations of *Camper*) longer of closing after birth than the left one.—*La Lançette Française.*

---

### MM. Rayer and Breschet on Glanders.

The Minister of War having recently addressed the Academy of Sciences on the subject of the great prevalence of glanders among the horses of the army, MM. *Breschet* and *Rayer* undertook a series of experiments and researches to determine the laws of its contagion and the mode of its transmission to man. They justly remark that, " as comparative anatomy and physiology have thrown much light on human anatomy and physiology, so the study of the diseases of the lower animals will in all probability contribute to dissipate many of the doubts and obscurities which still hang over many departments of human pathology."

Glanders, one of the most fatal diseases of the horse, has the baneful property of being communicable, alike by inoculation and by infection, to other animals, and strikingly to man himself. The truth of this has only within the last few years been proved beyond dispute. Even in the present day some veterinary surgeons continue to affirm that the disease is exclusively peculiar to solipedous animals; but in our opinion numerous indisputable observations and experiments have satisfactorily shewn that it is transmissible to man and some of the lower animals, as the dog, the sheep, and the goat.

It is however to be observed that it is only among solipedous animals that the disease has ever been observed to originate spontaneously : no authentic example of its original development in any ruminant or carnivorous animal has been recorded. For instance, in reference to dogs, they have been known to have lived with impunity for a length of time in stables where numerous glandered horses are kept; and yet it is equally well known that if they are directly inoculated with glanderous matter, they exhibit all the symptoms of the disease in the horse.

In the case of man, no instance of the spontaneous development of glanders has ever been observed ; but in all the instances on record the disease seems to have originated either by the direct inoculation of the glanderous matter into a wound, or by the infection of the atmosphere of stables where glandered horses were kept.

It seems that certain diseases are primarily peculiar to certain genera of animals, but that, when once developed, they are capable of being communicated to other genera. Such is the case with glanders; such also is the case with hydrophobia of the dog, and with the malignant pustule of ruminating animals. This peculiarity in the original development of some diseases deserves the notice of pathologists and naturalists; for if man does not spontaneously generate these maladies, the exemption certainly cannot be attributed to his not being exposed to the influences which are believed to engender them. Thus fatigue, imperfect and unhealthy nourishment, and the crowding together of numbers in a limited space, (to which causes veterinary surgeons usually attribute the spontaneous development of glanders,) have never been known to have given rise to the disease in man.

The disease of glanders may be either acute or chronic; it may either pass through its course in a few days, or continue for months or even years before it proves fatal. The acute disease is much more contagious than the chronic form; and it would even seem that the latter is by no means readily communicable, unless the disease from some cause or another be temporarily aggravated. The circumstance of the greater contagiousness of the acute than of the chronic form of glanders in the horse finds a parallel in the history of the syphilitic disease in man.

We know that blenorrhagia and chancre, in their chronic condition, are by no means readily communicable from one person to another, that the pus from old venereal sores is only feebly contagious, while that from consecutive sores is not so at all.

The circumstance of the acute and chronic forms of glanders differing so much in their contagiousness has had the unfortunate effect of inducing some veterinary surgeons not only to regard them as two distinct diseases, but also to question the contagiousness of the disease under any condition. The French veterinary surgeons have fallen into this error more than those of any other nation; and it is probably to this circumstance that we are to attribute the great prevalence of the disease in various parts of the country, more especially among the horses of our armies.

It is surely unnecessary to adduce proofs in the present day to shew that the disease is communicable from the horse to man. Independently of numerous well-authenticated cases reported within the last eight or ten years, we may state that we have succeeded in producing the disease in the horse and in the ass by inoculating them with the matter taken from glanderous pustules in the human subject. This experiment has been repeated by others, and has been attended with the same results.

It appears that the ass is more readily susceptible of the disease by inoculation than even the horse, usually developing itself in the former with unusual promptitude and intensity.

To shew the identity of the disease in the horse and in man, let us briefly review the characteristic or pathognomonic symptoms of the disease, as recognized by veterinary surgeons. They are three—the discharge from the nostrils, the engorgement of the submaxillary lymphatic glands, and the ulcerations of the nasal mucous membrane. Now we acknowledge that these three symptoms may be imperfectly manifested, in the human body, when infected: the discharge from the nostrils seems to be absent altogether in some cases. Perhaps however in these very cases the discharge may flow back into the fauces, while little or none escapes from the nostrils. Hence in glanders occurring in man, we frequently observe that there is an expectoration of mucus tinged with blood, whereas there is never any such symptom in the horse. Again, although the nasal mucous membrane may be affected in man, yet the enormous difference between the extent of the septum in man and in the horse may account for this symptom being so much less striking and conspicuous in the former.

As to the essential characters of the nasal eruption, and as to its seat and the nature of the morbid secretion in acute glanders, we do not hesitate to assert that they are identically the same in man and in the horse. The eruption is observed on the surface not only of the septum, but also of the spongy bones and on the posterior part of the velum palati; more rarely it occurs also on the anterior part of the velum and in the inside of the cheeks.

The engorgement of the submaxillary glands—a symptom which very generally exists in the horse—is of comparatively rare occurrence in the human subject. The absence of this symptom in the latter has been one of the causes that have led some to deny the identity of the disease, as it occurs in the horse. But it may be satisfactorily explained; for, independently of the much greater extent of the diseased nasal surface in the horse, it is found that there is a much more direct relation between the nasal fossæ and the lymphatic glands below the jaw in this animal than in the human subject.

As to the nature of the nasal eruption and the ulcerations which follow it, there is the most obvious identity. It must indeed be confessed, that when the eruption is scanty and limited in man, its traces are sometimes not very obvious after death, whereas in the case of a glandered horse they are always abundantly conspicuous.

There is often considerable difficulty in determining the existence of chronic glanders in man, which is not experienced in the case of solipedous animals.

Every horse, which is affected with a chronic discharge from the nostrils, with ulcerations on the septum or spongy bones, and with a thickening of the mucous membrane, accompanied with an engorgement of the submaxillary glands, is pronounced to be glandered; but in man, it is not sufficient to ascertain that there are ulcerations in the nostrils, and a more or less complete destruction of the septum (even with an engorgement of the submaxillary glands) to mark the case down as one of glanders. Every physician knows that this category of symptoms may exist in the human subject quite independently of any glanderous infection, and not unfrequently is the result of venereal and scrofulous disease. Hence it is always necessary to establish that such is not the case in a human patient, before we admit that the malady is of a glanderous nature.

If syphilitic or scrofulous ulcerations of the nasal passages can, up to a certain point, simulate the chronic glanders in man, on the other hand we have the certainty that the latter disease has in more instances than one been mistaken for a venereal affection.

In the small number of cases of chronic glanders observed in man, the swelling of the submaxillary gland has been so rarely noticed, that this very symptom, to which so much importance is attached by veterinary surgeons as indicative of the disease in the horse, must be considered in him as a proof rather of scrofulous than of glanderous contamination. Not only in several well-authenticated cases of chronic glanders in the human subject has the swelling of the glands been absent, but it likewise deserves notice that this is an almost constant symptom of syphilitic ozena accompanied with ulcerations of the throat. Besides being one of the most common of all the outward marks of a scrofulous diathesis, the enlargement of these glands is, every one knows, apt to be induced by various lesions of the lower jaw, eruptions on the scalp, ulcerations of the gums and cheeks, &c. &c.

The glanderous eruption has been observed both in man and in the horse; it seems to be, on the whole, more frequent in the former than in the latter.

As to the lobular pneumonia, which has been recognised as one of the lesions of the farcy glanders (morve farcineuse) in man, its existence as an element of the acute farcy glanders in the horse, after having been stoutly denied by many veterinary surgeons, has been within the last few years observed so frequently and so decidedly, that no longer any doubt can remain on this point. In this respect therefore the analogy is complete.

As to the lesions of the skin in acute glanders in man and in the horse, we are struck with this primary difference, that the development of pustules on the skin and of subcutaneous and intermuscular abscesses is of much more frequent occurrence in the former, than in solipedous animals. Moreover, in man, the eruption is usually rather indistinct on all the points of the surface of the body, (the face excepted, where it is generally most abundant,) whereas, in the horse, it appears most frequently on those parts free from hair, such as the circumference of the mouth, and the "fourreau." In dogs that have been inoculated with the virus of glanders, the scrotum has been sometimes found to be seized with inflammation and gangrene, while every other part of the body has remained exempt.

It is true, indeed, that in general, the acute glanders being regarded as so very infectious, and the diseased horses, in consequence, being usually killed before the malady has passed through all its stages, it is scarcely possible to determine with accuracy the relative frequency of the cutaneous eruption in them. Still we have sufficient data to warrant the assertion that it is less common in them than in the human subject.

It would seem, indeed, that the presence of thick hair upon any part of the body is an impediment to the formation of pustules or other eruption there. This we observe to be the case not only in horses affected with glanders, but also in cows affected with cow-pox, and in sheep affected with the malignant pustule.

In the acute farcy glanders, the cellular tissue and the lymphatic vessels which permeate it, become inflamed, and suppurate alike in man and in the horse. In both, infiltrations of purulent matter and deposits of plastic lymph are observed in the interstices of the muscular fibres; but the cellular tissue of the horse presents more rarely numerous and extensive abscesses than that of the human subject. Perhaps upon the whole the process of suppuration is more readily excited in man than in the lower animals.

In the horse as well as in man small deposits of pus have been found between the pericranium and the bones of the skull, and the latter have been several times observed to be in a carious condition. As to the relative frequency of osseous disease from farcy glanders in the human subject and in solipedous animals, it is scarcely possible to determine this point, for want of sufficiently numerous and accurate data. The study of these glanderous lesions of the bones is the more interesting as several physicians, from not being aware that they are known to occur in the horse, have not hesitated to attribute them, when they have been observed in men affected with the disease, to a syphilitic taint, even though there was not a single symptom to denote the existence of such in the patient. The fact of the same osseous lesions, recognised as of glanderous origin in the horse and attributed to syphilis when occurring in man, may be cited, among many other facts, as a proof of the advantages to be derived by the physician from a knowledge of comparative pathology.

Inflammation of the veins, and of the lymphatic vessels and glands, has been observed both in man and in the horse, and in nearly the same proportion. The same, perhaps, may be affirmed as to the relative frequency of circumscribed abscesses in the parenchyma of the liver, spleen and kidneys.

In fine, it may be confidently asserted, that the various lesions which have been observed in the acute and chronic glanders of the horse, have been met with in the acute and chronic glanders of the human subject. The differences which have been noticed, and to which we have alluded, are the less degree of nasal discharge, the occasional expectoration of it from the throat, the greater frequency of the pustular and gangrenous affection of the skin, and the rarity or total absence of the swelling of the submaxillary glands in man than in the horse.

The diagnosis of acute glanders in the human subject is not, in the present day, attended with greater difficulties or uncertainty than in solipedous animals. When the very existence of the disease was not recognised by medical men, and when they were not in the habit of examining the condition of the nasal passages after death, glanders in the human subject was most frequently confounded with malignant pustule.

But these two diseases differ from each other in many respects. In the former, the constitutional symptoms precede the eruption on the skin; whereas, in the latter, the carbuncular affection is primary and at first local; and in it we do not observe either the numerous farcy abscesses nor the characteristic ulcerations of the nostrils. In short, the acute form of farcy glanders, when it occurs in the human subject, is perhaps of all eruptive fevers that of which the diagnosis is the most easy: the truth of this assertion will appear from the circumstance of the disease having been at once recognised, and correctly too in every instance, in fifteen cases which have presented themselves within a short time in the hospitals at Paris.

In man the subcutaneous abscesses and a pustular and gangrenous eruption on the skin are often the earliest positive signs of glanderous infection, and these are well characterised before any ulcerations in, or discharge from, the nostrils can be detected. In the horse, on the contrary, the certainty of the diagnosis rests chiefly on the existence of a discharge from the nostrils, and of a pustular and gangrenous eruption in the nasal passages—the eruption being easily discovered in the septum by slightly opening the nostrils.

The diagnosis of chronic glanders is much more easy in the horse than in man. In fact, every case of chronic nasal discharge, attended with swelling of the glands in the horse—unless, indeed, these symptoms have been induced by the accidental introduction of a foreign substance into the nostrils, or by some cancerous affection of these parts—is at once considered to be of a glanderous nature. In such circumstances the veterinarian has not, like the physician, to ascertain if the nasal ulcerations are or are not of a syphilitic or a scrofulous origin.

Lastly, as to the treatment of glanders, whether in the human subject or in the horse, we are sorry to confess that as yet scarcely any progress has been made to the discovery of a cure. In an immense majority of cases, whether the disease be of the acute or of the chronic form, it proves fatal to the horse; and in man it has been uniformly fatal.

The leading and chief object must be to prevent, if possible, the development of the disease in the horse, by removing all the causes which may give rise to it, or which favor its propagation. Above all, let any doubts as to the contagiousness of the disease be for ever at an end; for, surely, no one who has once witnessed the ravages committed by the disease in stables, into which one glandered horse has been admitted, can question it for a moment. Neglect in this respect has been the chief cause of the frightful mortality among the horses of the army in various barracks throughout the kingdom. We trust that henceforth stricter and better precautions will be taken, not only in our military and public, but likewise in all private stables, to arrest the destructive progress of this pestilence.—*Gazette Medicale.*

On the preceding memoir M. *Magendie* took occasion, at a subsequent meeting of the Academy of Sciences, to comment in the following strain.

"I will tell my honourable confrere that, when he asserts that the chronic glanders is of the same nature as the acute malady, he is quite in error, and that he is equally mistaken in affirming that the former disease is contagious, or that the glanders of the horse is communicable to man by the way of contagion.

With respect to the first of these points,—the difference between the acute and chronic forms of glanders—the chief distinguishing marks are these: the acute disease usually runs its course rapidly, and from its very commencement compromises the life of the animal, rendering it quite incapable of any exercise; whereas in the chronic disease the horse is in most instances fit for work, and not only takes its food well, but will propagate its race, as if it were in good health, for a series of months and even of years. Indeed, there are so few grounds to confound the two diseases, that they may actually co-exist in the same animal, and the veterinarian can distinguish what appertains to the one and what appertains to the other.

With respect to the contagiousness of the chronic disease, I may state that the commission, recently named by the Minister of War to examine minutely into the history of glanders generally, made numerous experiments to determine whether healthy horses ever become infected by being kept in the same stables with such as were diseased. Now although this proximity was sometimes continued for upwards of a twelvemonth, in no instance had we the slightest reason to suspect the communication of the chronic disease from one animal to another."

M. *Magendie* is equally sceptical as to the transmissibility of equine glanders to the human subject.

" . . . . . . For my part, I, who have studied the disease in the horse on a wide scale, and have seen persons affected with the malady which my confreres denominate glanders, must confess that I have never discovered the supposed resemblance, and should have been most happy if it could have been pointed out to me."

It would seem, nevertheless, that M. *Magendie* has been puzzled what to make of the disease, which, he cannot deny, is apt to seize persons who are brought much in contact with glandered horses.

" If," says he, " I may judge by my own observations of this disease, which I regard as *new,* it is probably of a carbuncular nature, and is dependent upon a morbid state of the blood."

Several of the Academecians replied to M. *Magendie,* who seems to stand very much alone* in his negation of most of the positions laid down in the memoir of MM. *Rayer* and *Breschet.*

The following facts were mentioned by one gentleman on the authority of M. *Leblanc,* one of the most distinguished veterinary surgeons in Paris.

1. Pus obtained from the pustules on a man affected with glanders, and also the nasal mucus of the same patient were inoculated upon an ass: the animal speedily exhibited all the symptoms of genuine glanders and died. The correctness of this fact was ascertained by several eminent veterinarians.

2. A quantity of the same pus and mucus was inserted in a horse; in the course of a few days glanderous and farcy lesions presented themselves, and the animal was killed at the end of a month. On dissection, all the morbid appearances usually observed were found.

In conclusion, we may state that a commission, consisting of MM. *Pariset, Juge, Emery, Huzard,* and *Guarard,* recently sent to the head Prefect of Police their report, wherein, among other regulations, we find the following.

1. " It is prohibited to any one to sleep, or to cause another to sleep, in a stable where there are any horses suspected to be glandered."

4. " Every person who may have in his possession a horse, mule, or ass affected, or suspected to be affected, with the glanders or the farcy, shall be bound to make immediate declaration of the same, in rural districts before the mayor, and at Paris before a commissioner of police."

15. All stables or buildings where horses or other animals affected, or sus-

---

* We have subsequently discovered that M. *Larrey* is not satisfied as to the transmission of the glanders from the horse to the human subject. He alluded to his experience during the protracted continental war, carried on by France for a quarter of a century, during the whole course of which he had never heard of any horse-soldier having ever caught the disease. Without, however, denying the possibility of inoculation, he is of opinion that further enquiries are necessary before we can admit its perfect truth.

pected to be affected, with the glanders or the farcy shall be well ventilated and purified by the orders of the mayor or commissioner of police; and they shall not again be occupied until it has been ascertained by an expert veterinary surgeon that the causes of infection no longer exist.—*Gazette Medicale.*

---

### On the Structure and Connections of the Placenta.

As the following description and observations are the result of most carefully conducted anatomical investigations, they will be read with interest.

M. Bonamy says, "a woman died in between the seventh and the eighth months of pregnancy. An injection of size coloured with red lead was thrown with great care into the venous system of the uterus from the common iliac vein and one of the ovarian veins. A second injection, composed of the essence of turpentine and coloured with indigo, was then thrown into the uterine arteries from the extremity of the aorta, ligatures having previously been placed upon all the vessels capable of permitting the injection to pass to the lower limbs. The uterus was carefully removed from the body and conveyed to the lecture-room of M. *Cruveilhier.* The uterus was then laid open at some distance from the attachments of the placenta, and the fœtus removed. We immediately observed that the umbilical vessels on the placenta and along the entire extent of the cord were distended with a black fluid, which we imagined was the dark turpentine injection thrown into the uterine arteries. But we soon discovered our error; for on dividing the cord nothing but blood escaped.

Injections, consisting of linseed oil coloured with white lead and with yellow ochre, were thrown into the umbilical vein and then into one of the umbilical arteries.

Already we perceived on the fœtal surface of the placenta the red fluid which had been injected into the uterine veins; and the question naturally suggested itself—By what channels had it reached so far? To solve this, it was necessary to examine with great care the nature of the connexions of the placenta with the uterus.

We carefully detached a small portion of it by cutting with the greatest precaution first the *decidua,* and then the ligamentous bridles which attached it to the inner surface of the uterus: we could thus examine the utero-placentary connecting tissue and the vessels which it might contain.

This tissue is of an albuminous or fibrinous nature, and is formed of numerous lamellæ which cross and intersect in all directions, and adhere to each other only by a few points of their surface.

If air is blown in at the places where they are slightly apart (où elles ne sont que contiguës) we observe that cells of various forms and sizes quickly make their appearance. These cells having been seen by several anatomists developed by the injection which had been thrown into the uterine veins, it has been a general belief that they are actually veins which communicate freely with the uterine system of veins.

An author to whose authority we have always confidence in appealing, M. *Velpeau,* has however dissented from this doctrine; and "in eight cases in which I examined the placenta *in situ* (he says), I have not been able to discover any sinuses or orifices which bore any resemblance to what have been described by writers under these names."

M. *Jaquemier,* in his recent work on the uterus published last year, has described with the greatest minuteness these cells under the title of veins, and has gone so far as to distinguish three principal varieties of them. One set, he says, is extremely short, (their *trajet* not exceeding one or two lines), and is not very oblique in their direction; whereas, another set is very obliquely or slantingly arranged, and is most frequently situated in the direction of the inter-lobular scissures; the last set has the most remarkable distribution, being arranged in the form of a crown or circle around the placenta.

All these so-called veins communicate, according to M. *Jaquemier*, with the uterine veins by large orifices on the internal surface of the uterus where the placenta is attached. We must confess that we have not been able to discover these large orifices of communication described by this author; and in our experiment the cells did not contain a particle of the injection which had been thrown into the uterine veins. We first blew air into them, then we filled them with water; but neither the one nor the other of these fluids seemed to penetrate. (To render these experiments more decisive, we had previously freely exposed the uterine veins by dissecting off a flap from the outer surface of the uterus.) These alleged orifices in truth do not exist; we regard them as mere solutions of continuity produced most frequently by the injection having been thrown in with too much force. The materials commonly employed have been suet or wax, neither of which, it is well known by anatomists, is at all suited for the injection of the veins: in consequence of the rapid cooling and solidification of these substances, it is necessary to throw the injection in hurriedly and with considerable force. Hence rupture of some of the veins almost inevitably takes place; this is especially apt to happen with the injection of the uterine veins, during the state of pregnancy, in consequence of their parietes having become much distended and consequently weakened. When therefore we examine the attachment of the placenta to the gravid uterus, after such an injection has been used, we almost inevitably find some of the injected matter, like small lumps, in cavities the orifices of which, although communicating with the veins, are in truth nothing but ruptures.

The utero-placentary tissue adheres most intimately not only to the walls of the uterus, but also to the large veins which are distributed on its internal surface. In detaching the placenta, the rupture of these adhesions is sufficient in itself to cause the rupture of the parietes of the veins; but in truth there is more than these adhesions between the uterine veins and the utero-placentary tissue, for there is a "veritable continuité" established by minute-blood-vessels which may be observed imbedded in the substance of the adhering lamellæ. These minute vessels are the genuine utero-placentary veins. Now when the adhering lamellæ are lacerated in detaching the placenta from the uterus, these vessels are necessarily torn across at their embouchure into the large veins, the rupture of which also is generally occasioned at the same time.

In the first portion of placenta which I detached, I did not perceive the orifices of communication, in consequence no doubt of the adhering utero-placentary tissue having been cleanly divided with a sharp scalpel, and not torn across. But the same precaution being not used in detaching the next portion—which was separated with the fingers—the ruptures of the uterine veins were at once made obvious by the escape of the injected fluid.

Now if it can be shewn that the cells in the utero-placentary tissue do not communicate with the veins, it follows that the appellation of utero-placentary veins, which has been given them by many writers, is quite misapplied, and gives rise to a most erroneous opinion as to their real nature. The parietes of these cells are very unresisting, and yet they must contain a fluid in circulation. It is true that the blood is stagnant in them as in a lake, to use the expression of *Haller;* but when the contractions of the uterus come on during labour, this blood will be set in motion and will re-act against their parietes, which would readily give way, and then a hæmorrhage more or less copious would necessarily follow, if it were not that the uterus was distended with its contents, and thus a uniform pressure is kept up at every point of its internal surface.

We shall now make a few remarks on the small vessels which we have described to be imbedded in the lamellæ of the utero-placentary tissue, and which transmit the venous blood of the uterus into the placenta—in other words, the genuine utero-placentary vessels. The veins are of about the same size as the arteries, and sometimes rather larger; some that I measured had a diameter of two or three lines. The veins were easily distinguishable from the arteries, by the colour and nature of the injection within them, and which consisted of what had been thrown into the uterine veins: they were rectilinear; and their anastomoses, which were very frequent, formed extensive plexuses on the parietes of the cells. These plexuses penetrated at every point into the uterine surface of the placenta; their terminations in the large uterine veins were quite manifest. On the other hand, the arteries were arranged spirally, and their anastomoses were comparatively few; they were less numerous at the circumference than at the centre of the placenta, the entire thickness of which was penetrated with them. These arteries were distinctly observed to be continuous with the arteries of the uterus.

Besides these blood-vessels, some writers have described lymphatic vessels in the placenta. *Lanth,* in his memoir on the connections of the placenta, has admitted the existence of these vessels in the following passage:—" Since there is no direct communication between the blood-vessels of the uterus and those of the placenta, and since the cells where, it is has been alleged, the blood might be effused actually do not exist, the only communication therefore which we can admit between the mother and the fœtus is that by lymphatic vessels, of which some terminate in the vessels of the placenta and others in those of the deciduous membrane. These vessels (the lymphatic), by their termination in the sanguineous vascular system of one of these organs, appear to be grafted at their origin on those of the other, in such a manner that the vessels, which spring or originate from the vessels of the uterus, and which terminate in the vessels of the placenta, draw from the maternal blood certain materials to be conveyed into the circulation of the fœtus:—on the other hand, the lymphatic radicles, which are grafted on the blood-vessels of the placenta, terminate in the vessels of the uterus, for the purpose of withdrawing from the fœtal blood such matters as are no longer useful to it, and excreting them into the venous system of the mother."

M. *Breschet* has very justly remarked that the above explanation is all very ingenious, but that it is quite unsupported by any anatomical facts.* . . . . The vascular connexions of the placenta with the uterus in the human subject are altogether analogous with those observed in the lower animals. We find in the former the veins and the arteries which we have had occasion to describe—(M. Bonamy had in the previous part of his memoir described the anatomy of the placenta in the carnivorous, ruminant, and pachydermatous animals)—under the name of the maternal vessels of the placenta. We now proceed to describe those which belong to the fœtus, under the name of the umbilical vessels.

The umbilical arteries and veins, on reaching the fœtal surface of the placenta, divide into numerous large branches, which are situated under the amnios and chorion. The arterial branches communicate freely with each other in the substance of the same cotyledon. If we throw even a coarse injection into one of the arteries, it returns immediately by another: if we continue to push more in, the injection is found to pass from the arteries into the veins. But if we inject from the veins, the injection does not pass, except with much difficulty, into the arteries. This impediment to the transmission from the veins into the arteries cannot well be attributed to the presence of valves in the former; for they have not been discovered by some of the best anatomists of the present day. If a fine injection be thrown into the veins, the uterine surface of the placenta will

* It is right to state that M. *Lanth* himself, in the last Edition of his Manual of Anatomy, has recanted his former opinion, and expressly admitted that no intermediate lymphatic vessels, such as he had described, can be shewn.

be converted into a close vascular net-work, from which however none of the injected fluid will escape. *Ræderer* had already remarked that no blood flows from the uterine surface of the placenta, when it has been just extracted from the uterus, and that none can be forced from it even by pressure. When a placenta, which has been minutely injected, is macerated for some time, we find that it becomes resolved into flocculi covered at numerous points with a soft pulpy substance, which is not easily detached. These flocculi, when examined with a magnifying-glass, exhibit numerous granulations or *acini*, composed of minute convoluted vessels, in the manner of the vessels of the chorial villosities in the cow and sheep. If the maceration be prolonged, the small vessels lengthen out and lose most of their tortuosity.

If we compare the vascular elements which enter into the composition of the human placenta, we see that they are of two kinds, as in the lower animals—viz. the maternal and the umbilical vessels.

In examining the structure of the placenta in ruminant animals, we have seen that the maternal vessels form vast plexuses in the substance of the cotyledons, and then that the vascular filaments of the chorial villosities become blended and entangled among these plexuses. Now the strictest analogy exists between this arrangement and that which we discover in the placenta of the human subject.

Each of its cotyledons is constituted in the following manner; the maternal or utero-placentary vessels penetrate it at all the points of its uterine surface, and form in its substance excessively close net-works; the umbilical vessels, which penetrate it from the fœtal surface towards the uterine surface, exhibit the same arrangement as the vessels of the chorial villosities, under the form of granules or *acini;* and they are convoluted upon themselves among the meshes of these net-works.

But here, as in carnivorous animals, it is impossible to separate the vessels which belong to the mother from those which appertain to the fœtus. The connexion which exists between these two orders of vessels appears to us to result from the membranous sheath which continues to envelop them even in the very substance of the placenta. This sheath is supplied to one set from the chorion, and to the other from the lamellar prolongations of the utero-placentary tissue.

The maternal vessels cannot be traced so distinctly as in the placenta of ruminant or pachydermatous animals, since it is not possible to separate the umbilical vessels from them; still they are visible to the naked eye. It is the veins chiefly which form the networks which we have described above; the arteries seem not to penetrate so far into the substance of the placenta.

But this difference may be only apparent, and may arise from the injection of the arteries having been not so perfect as that of the veins.

It follows from the preceding remarks, that no direct communication between these two orders of vessels which constitute the placenta in the human subject can be discovered.

A fine injection thrown into the uterine veins is found to penetrate even to the fœtal surface of the placenta, (as in the lower animals,) the maternal vessels extending their ramifications through its entire thickness; but yet we are not able to say that these two orders of vessels—the uterine and the fœtal—communicate with each other; but an injection thrown in from the umbilical cord is not found to penetrate into the uterine veins.—*Gazette Medicale.*

